# Estimates of global burden of snakebite: A literature review and geostatistical modelling study

**DOI:** 10.1371/journal.pmed.1005201

**Published:** 2026-08-25

**Authors:** Dileepa Senajith Ediriweera, Anuradhani Kasturiratne, Arunasalam Pathmeswaran, Ymkje Stienstra, Gerardo Martín, Takuya Iwamura, Kris A. Murray, Hithanadura Janaka de Silva, Peter John Diggle, David Griffith Lalloo

**Affiliations:** 1 Centre for Snakebite Research and Interventions, Liverpool School of Tropical Medicine, Liverpool, United Kingdom; 2 Health Data Science Unit, Faculty of Medicine, University of Kelaniya, Ragama, Sri Lanka; 3 Department of Public Health, Faculty of Medicine, University of Kelaniya, Ragama, Sri Lanka; 4 Department of Internal Medicine/Infectious Diseases, University of Groningen, University Medical Centre Groningen, Groningen, the Netherlands; 5 Kenya Snakebite Research & Intervention Centre, Kenya Institute of Primate Research, Ministry of Health, Nairobi, Kenya; 6 Departamento de Sistemas y Procesos Naurales, Escuela Nacional de Estudios Superiores unidad Mérida, Universidad Nacional Autónoma de México, Mérida, Yucatán, México; 7 Department F.-A. Forel for Aquatic and Environmental Sciences, Faculty of Science, University of Geneva, Geneva, Switzerland; 8 Centre on Climate Change and Planetary Health, MRC Unit The Gambia at the London School of Hygiene and Tropical Medicine, Fajara, The Gambia; 9 Department of Medicine, Faculty of Medicine, University of Kelaniya, Ragama, Sri Lanka; 10 CHICAS, Lancaster University Medical School, Lancaster, United Kingdom; Lao-Oxford-Mahosot Hospital-Wellcome Trust Research Unit, LAO PEOPLE'S DEMOCRATIC REPUBLIC

## Abstract

**Background:**

Snake envenoming (SE) remains an important public health problem, disproportionately affecting impoverished rural communities in tropical and subtropical regions. Although previous attempts have been made to quantify the global burden of SE, accurate, up-to-date data remain scarce. Here we present a comprehensive re-evaluation of the global burden of snakebite, 17 years after our initial estimate.

**Methods and findings:**

A new literature review was conducted on snakebites, SE and mortality published in any language, up to March 31, 2025, to update the knowledge accumulated since our first global burden estimate. Based on country- and region-level aggregated data, and hospital and community-based survey data, we modelled SE and mortality burden in relation to geographic location. Explanatory variables were selected to reflect the underlying social and natural environmental conditions. *Low* and *high* estimates of the global snakebite burden were calculated using geostatistical models that produced individual-country estimates, accounting for possible underreporting of data when appropriate.

We estimate that at least 2.1 million envenomings and 274,000 deaths occur globally each year due to snakebites, and our *high* estimates suggest up to 7 million envenomings and 513,000 deaths annually. Low-income countries exhibit the highest incidence and mortality rates, with sub-Saharan Africa accounting for 45% of global envenomings and deaths, nearly three times the incidence observed in South Asia. These estimates suggest that previous burden estimates could substantially underestimate the scale of the problem.

The main limitations of this study are the scarcity and heterogeneity of empirical data in many countries and the reliance on modelling assumptions to estimate burden in settings with limited or no direct observations.

**Conclusions:**

Snake envenoming is a significant cause of morbidity and mortality globally, with sub-Saharan Africa, South Asia, and Southeast Asia being the most severely affected regions. Further improvement in the quality of epidemiological data is required for more accurate estimates of the global burden, particularly in sub-Saharan Africa.

## Author summary

**Why was this study done?:**

- Snake envenoming causes substantial illness and deaths worldwide, particularly in poor rural communities in tropical and sub-tropical regions.
- Existing estimates are based on limited and dated data and are likely to underestimate the true burden of snakebite.
- Updated estimates are needed to understand the global burden of snakebites, guide prevention and treatment efforts, improve access to care, and support global efforts to reduce snakebite-related illness and deaths.

**What did the researchers do and find?:**

- We reviewed published literature on snakebites, snake envenomings and related deaths from around the world up to March 2025.
- We used geostatistical models to estimate the incidence and annual number of snake envenomings and deaths in countries with snakes, including those with limited or no data.
- We estimate that at least 2.1 million people experience snake envenomings and 274,000 people die each year, with higher estimates suggesting up to 7 million envenomings and 513,000 deaths annually.
- The highest envenoming incidence and mortality rate is observed in low-income countries, particularly in sub-Saharan Africa, South Asia, and Southeast Asia.

**What do these findings mean?:**

- Snake envenoming is a major global health problem and may affect substantially more people than previously recognised.
- These findings highlight the need for improved prevention, surveillance, access to antivenom and clinical care, particularly in the most affected regions.
- These updated estimates can help governments and international organisations prioritise resources and monitor progress towards reducing the burden of snakebite.
- Our estimates are limited by the availability and quality of data in many countries, and countries with little or no direct information have greater uncertainty in their estimates.

## Abstract 3

In this modeling study, Dileepa Ediriweera, Anuradhani Kasturiratne and colleagues reviewed the literature on snakebites, and used this data to estimate the incidence of annual number of snake envenomings and deaths in countries with snakes.

## Introduction

Snake envenoming (SE) mainly affects resource-poor, rural agricultural communities in tropical countries, where it results in a high medical and economic toll [1]. Many survivors of snakebite experience disabilities and long-term health consequences, which negatively impact their quality of life [2,3]. Recognition of the scale of the problem led to WHO including snake envenoming in its list of Neglected Tropical Diseases in 2017 [4]. However, the true global burden of snake envenoming, its impact, and distribution within different regions remain poorly characterised. Such information is essential for allocating limited healthcare resources, particularly antivenom and intensive care facilities, and training healthcare professionals to treat snakebites.

Although snake envenoming is acknowledged as a major public health problem, reliable incidence and mortality data from the rural tropics where snakebites occur most commonly are scarce. Most estimates of snakebite are based on hospital returns or incomplete central databases. Bites and associated mortality are underreported because many victims do not seek treatment in standard health facilities, preferring traditional treatment [5–9]. The incidence of snakebites also varies geographically and seasonally [6,10–12]. Regional variations in incident snakebite deaths have been demonstrated in a previous nationwide survey conducted in India [10]. This type of regional variation is seen even in small countries such as Sri Lanka [13]. In the face of such heterogeneity, extrapolating the results of small local surveys is fraught with inaccuracies [9]. To address this problem, population-based studies of incidence and mortality from countries that appear to have the highest caseloads and mortality are urgently required; this is especially the case in sub-Saharan Africa and Southeast Asia.

Following the reviews by Swaroop and Grab in 1954 [14] and Chippaux in 1998 [15], a global burden review in 2007 estimated 1.2 to 5.5 million bites, 420,000 to 1.8 million envenomings, and 20,000–94,000 deaths due to snakebite each year, with South Asia, Southeast Asia and sub-Saharan Africa being the most affected. [16]. However, the inadequacy of available data and the consequent need to rely on extrapolation meant this estimate was far from perfect. This was shown in two later studies: a review of snakebite burden in sub-Saharan Africa, which estimated 314,000 envenomings and 7,300 deaths annually (comparable to the 2007 global estimates), though household surveys indicated that incidence and mortality were likely to be 3–5 times higher [2]; and a study from India that estimated snakebite deaths to be three times the estimate reported in the global burden review [10].

Since 2008, there has been a welcome increase in the snakebite literature [17,18], with several good-quality epidemiological studies. This, together with WHO’s inclusion of snakebite as a Neglected Tropical Disease, has resulted in a rise in interest among scientists, policy planners, international health organisations, non-governmental organisations, and funding agencies. We have re-estimated the global burden of snakebite, by reviewing the currently available literature on snakebite worldwide and developing a scientifically robust and replicable method for burden estimation, which could also be used for future updates.

## Methods

### Data sources

#### Epidemiological data.

The epidemiological data were identified through a literature search strategy targeting publications on the country or regional burden of snakebite, published in any language, between January 1, 2007, and March 31, 2025. To identify relevant publications, we searched the PubMed database using the terms (snakebites[MeSH Terms]) AND (incidence OR count OR number OR rate OR epidemiology OR morbidity OR mortality OR deaths OR statistics) and Scopus database using TITLE-ABS-KEY((“snakebite” OR snake bite OR “snake envenoming” OR “snake envenomation”) AND (incidence OR count OR number OR rate OR epidemiology OR morbidity OR mortality OR deaths OR statistics)). PubMed and Scopus yielded 1,689 and 3,757 publications, respectively. After merging 1,454 duplicates, 3,992 papers and book sections remained. Two investigators independently screened the abstracts of the research papers and excluded 3,377 irrelevant papers. We retrieved the full papers and relevant sections of the remaining 615 articles through PubMed, Google Scholar, ResearchGate, specific journals’ websites, and local and international library resources. Two investigators independently reviewed the content of the papers and their reference lists to identify publications with relevant data (excluding case reports and case series), resulting in 174 papers being extracted into a Microsoft Excel spreadsheet. A panel of senior investigators (DGL, HJdeS, and AP) reviewed the database and excluded the publications that had:

(a) cited data from another source/ publication that had been already identified and included in the database
(b) cited data from an older source/ publication that had been considered in our previous estimates of the global burden of snakebite
(c) cited estimates without describing the methods of arriving at the estimate adequately or described methods which are not scientifically sound
(d) directly cited numbers that were based on the estimates we made for countries without data in our previous (2007) estimates of the global burden of snakebite

This process ultimately resulted in 84 publications that provided robust estimates of snakebite, envenoming and mortality.

We also conducted web-based searches for grey literature originating from other sources, such as, National Ministries of Health, National Poison Centres and health agencies to identify reliable data that could supplement the data from scientific publications, leading to the inclusion of six web sources. These were from the National Ministries of Health and National Poison Centres for Thailand, Malaysia, India, Indonesia and Guyana and a publication from the Centers for Disease Control and Prevention (CDC) for the United States.

This resulted in a total of 90 data sources for the global burden estimations [13,19–106] (see S1 Appendix). If sources only provided overall snakebite data, we assumed that 50% of all snakebites lead to envenoming, based on a recent systematic review and meta-analysis, and supported by expert opinion [107]. We did not impute missing outcome data, and all available outcome data from the included sources were used in the analysis.

#### Socio-demographic and environmental data.

To reflect the complex interplay of ecological and socioeconomic determinants of snakebite risk, we selected the following multidimensional explanatory variable groups: snake richness, socioeconomic, demographic, climate, environmental, agricultural, and farming-related variables (Table 1) [108]. Country-level socioeconomic data were obtained from the United Nations Development Programme for the years 2005, 2010, 2015, and 2020 [109]. The share of land area used for agriculture in 2005, 2010, 2015, and 2020 was obtained from the Food and Agriculture Organisation of the United Nations, via the World Bank (2025) [110]. The share of the population living in urban areas for 2005, 2010, 2015, and 2020 was obtained from the United Nations Population Division, via the World Bank (2025) [111]. Spatially continuous surfaces of monthly minimum temperature, maximum temperature, and precipitation with 5 km x 5 km resolution from 2000 to 2024 were obtained from WorldClim [112]. We considered monthly climate data to compute annual summary measures, including the average, maximum, and standard deviation of maximum temperature; the annual average, minimum, and standard deviation of minimum temperature; and the average, total, and standard deviation of precipitation. Elevation data with a 5 km x 5 km resolution was obtained using the elevatr package in R [113]. Population count data (i.e., Unconstrained global mosaics at 1 km resolution) for 2005, 2010, 2015, and 2020 were obtained from WorldPop [114]. Population counts were rescaled to match a 5 km x 5 km resolution and used in the analysis. The richness of Category 1 snakes (i.e., widespread, medically important species causing significant morbidity, disability, or mortality) and Category 2 snakes (i.e., species capable of causing morbidity, disability, or mortality, but less frequently implicated or lacking sufficient epidemiological or clinical data), with a 5 km x 5 km resolution, was obtained from a previous publication [18]. For the analysis, we considered both the maximum (i.e., highest richness value among grid cells) and the mean (i.e., average richness across grid cells) species richness of Category 1 and 2 snakes.

**Table 1:** Socio-demographic and environmental data considered for the analysis.

Domain	Variable	Resolution	Source
Health and Demographics	Life Expectancy at Birth for the overall population and by gender	Country-level	United Nations Development Programme for the years 2005, 2010, 2015 and 2020
	Adolescent Birth Rate		
	Child mortality		
	Maternal Mortality Ratio		
	Nutrition		
Education	Years of Schooling and Expected Years of Schooling for the overall population and by gender	Country-level	United Nations Development Programme for the years 2005, 2010, 2015 and 2020
	School attendance		
	Percentage of the population with at least some secondary education		
Economic and Social Status	Gross National Income Per Capita and Labour force participation rate for the overall population and by gender	Country-level	United Nations Development Programme for the years 2005, 2010, 2015 and 2020
	Share of seats in parliament by gender		
	Assets		
Inequality and Development	Human Development Index, Inequality-adjusted Human Development Index	Country-level	United Nations Development Programme for the years 2005, 2010, 2015 and 2020
	Gender Development Index		
	Gender Inequality Index		
	Coefficient of human inequality		
	Difference from HDI value		
	Inequality in education		
	Inequality in income		
	Inequality in life expectancy		
	Overall loss		
Environmental	Carbon dioxide emissions per capita	Country-level	United Nations Development Programme for the years 2005, 2010, 2015 and 2020
and Sustainability	Material footprint per capita		
	Planetary pressures adjusted Human Development Index		
Poverty	Multidimensional Poverty Index	Country-level	United Nations Development Programme for the years 2005, 2010, 2015 and 2020
	Access to Cooking fuel		
	Drinking water		
	Electricity		
	Sanitation		
	Housing		
	Years of schooling		
Agriculture	Share of land area used for arable agriculture	Country-level	Food and Agriculture Organization of the United Nations, via the World Bank for the years 2005, 2010, 2015 and 2020
Population	Share of the population living in urban areas	Country-level	United Nations Population Division, via World Bank for the years 2005, 2010, 2015 and 2020
Climate	Monthly minimum temperature	5 km x5 km resolution	WorldClim
	Monthly maximum temperature		
	Monthly precipitation		
Environment	Elevation	5 km x5 km resolution	elevatr package in R
Snake richness	Category 1 and 2 venomous and medically important snake species	5 km x 5 km resolution	Longbottom and colleagues, 2018
Population counts	Unconstrained global mosaic	1 km x 1 km resolution	WorldPop for 2005, 2010, 2015 and 2020

The datasets obtained from the United Nations Development Programme, Food and Agriculture Organisation of the United Nations, and United Nations Population Division were only available for the years 2005, 2010, 2015, and 2020. Therefore, we used the closest available year to match the epidemiological data for each country. Details of explanatory variables are provided in the S2 Appendix.

### Statistical methods

#### Exploratory analysis:

Incidence and mortality data were compiled from multiple data sources: country-level aggregated data, regional-level aggregated data, hospital-based data, and community-based survey data. Hospital records and national databases are prone to underreporting [8], and community-based surveys have demonstrated greater accuracy in capturing incidence and mortality, particularly in rural and underserved populations [13,85]. To address these data disparities, we incorporated data source type (i.e., country-level aggregated data, regional-level aggregated data, hospital-based data, and community-based survey data) as an explanatory variable in our modelling framework, allowing us to adjust for systematic biases inherent to different reporting methods.

Country-level explanatory variables were directly considered for the modelling, as these do not vary within geographical regions. When an explanatory categorical variable was missing for a given country, we imputed its value using the average of neighbouring countries, assuming their regional similarity. To evaluate the potential influence of these imputations on model estimates, we conducted sensitivity analyses excluding all countries with imputed covariate values (i.e., six countries for envenomings and four for mortality). The results of these analyses were used to assess the robustness of the model estimates and overall conclusions.

The spatially continuous, grid-level, explanatory variables that vary within geographical regions were converted to average values over the relevant areas (country or region), and these average values were used in the analysis. For community surveys and hospital data, we extracted the values at the centroid of each location and calculated averages over multiple buffer radii (10, 20, 50, 100, 250, 500, and 1,000 km). After assessing the log-likelihood ratios of these different average values, we selected bilinearly interpolated values for modelling community surveys and hospital-based data.

In the exploratory analysis phase, we ignored spatial correlation and used Poisson log-linear models [115] for each of the two outcomes of interest: envenoming bite incidence and mortality. We used empirical log incidence plots to visually examine the functional form of associations between explanatory variables and outcomes. We then used the corresponding generalised additive models [116] to check for any non-linear associations of explanatory variables with envenoming incidence.

The main objective of this analysis was to develop continuous risk surfaces for envenoming bites. As our objective was to predict the burden rather than estimate covariate effects, the explanatory variables with the greatest ability to explain the variability in envenoming bites were prioritised for model building. To structure our modelling approach, we grouped the explanatory variables into three categories: climatic, socio-economic and other environmental variables. We conducted explanatory analysis within each group to identify variables associated with envenoming incidence. These selected variables were then incorporated into a single non-spatial multivariable model. We investigated interactions between variables. To further explore spatial heterogeneity, we introduced landmass (i.e., a factor variable denoting eastern and western landmasses) into the model and evaluated the importance of landmass and of potential interactions with the explanatory variables.

Snakebite deaths form a subset of envenoming cases; however, not all envenoming data sources provided mortality data, and vice versa. Furthermore, the ratio of envenoming to deaths is likely to vary across regions due to differences in the composition of snake species, access to healthcare, and the availability of treatment. Therefore, we developed separate models for snake envenoming and mortality. Since snakebite deaths are related to envenoming incidence, we considered the estimated envenoming bite incidence as an explanatory variable for model building. In all other respects, we used the same methodology for both envenoming incidence and mortality.

#### Assessing spatial correlation:

Both non-spatial Poisson log-linear models showed overdispersion (envenomings: residual deviance = 451,583.73, df = 228, *P* < 0.001; mortality: residual deviance = 7,110, df = 161, *P* < 0.001). We therefore fitted mixed-effects Poisson log-linear models incorporating country-level random effects. The country-level random-effect variance was strongly supported in both models (Δ deviance 111,635.5 for envenoming and 4,682.63 for mortality). Accounting for the nonstandard null distribution for variance components [117], the evidence for between-country heterogeneity remained highly significant (*P* < 0.001 in both models).

To check for residual spatial correlation, we used the empirical variogram of the standardised Pearson residuals of each nonspatial model. In both cases, the variogram indicated the presence of spatial correlation. For our final models, we therefore used a model-based geostatistical approach.

#### Geostatistical modelling.

We adopted a model-based geostatistical framework to estimate envenoming bite incidence and snakebite mortality at each location. This approach accommodates residual variation arising from both spatially structured and unstructured heterogeneity through a latent Gaussian process. The nugget effect of the latent Gaussian process captures unexplained measurement errors, nonspatial sources of noise, and micro-scale spatial variation occurring at a finer scale than the spatial resolution of the data [118].

Parameter estimates, incidence mapping and spatial predictions for snake envenomings and for mortality were obtained from Poisson log-linear geostatistical models, as follows. At location *x*_i_, *Y*_*i*_ is the number of events (envenomings or deaths) out of *n*_*i*_ individuals at risk, where *n*_*i*_ corresponds to the number sampled, the underlying population of the corresponding administrative unit, or the denominator implied by the published incidence rate when not explicitly reported. The random variables *Y*_*i*_ are independent Poisson random variables conditional on a Gaussian process *S(x)* and independent Normally distributed random variables *Z_i_* with mean zero and variance *τ*^*2*^. The conditional expectation$\lambda \left(x_{i}\right),\;$of *Y*_*i*_ is given by


$$\begin{array}{c} log\left\{\lambda \left(x_{i}\right)\right\}=\text{log}\left(n_{i}\right)+d{\left(x_{i}\right)}^{T}\beta +S\left(x_{i}\right)+Z_{i} \end{array}$$ (1)

where *d*(*x*_*i*_) is the set of explanatory variables associated with location *x*_*i*_. The Gaussian process *S*(*x*) has mean zero, variance *σ^2^* and correlation structure Corr[*S(x), S(xˊ)*] = exp(*-u/*φ), where *u* is the distance between *x* and *xˊ* and *φ* determines the scale of the spatial correlation. The terms *Z*_*i*_ and *S*(*x*_*i*_) in (1) capture non-spatial and spatially correlated components of the residual spatial variation in *Y*_*i*_ after adjusting for the covariate effects.

Separate geostatistical models were fitted for envenoming bite incidence and snakebite mortality incidence. Initial values for regression coefficients and covariance parameters were obtained from the respective nonspatial generalized linear models and least-squares fits to the empirical variogram, respectively. A Markov chain Monte Carlo (MCMC) algorithm was used to simulate the samples required for Monte Carlo maximum likelihood estimation. Autocorrelograms and trace plots were assessed to check convergence. Both models demonstrated strong MCMC convergence, with large effective sample sizes for the envenoming incidence model (mean 2,165; range 1,651–2,853) and mortality incidence model (mean 1,920; range 1,494–2,393). Gelman-Rubin statistics [119] were consistently close to 1 for both the envenoming incidence model (mean 1.0008; range 0.9999–1.0046) and the mortality model (mean 1.0008; range 0.9999–1.0052), indicating low autocorrelation, good chain mixing, and satisfactory convergence.

#### Assessing goodness of fit:

Model validation used Monte Carlo methods based on variograms. For each of the two outcomes (envenoming incidence and mortality), we simulated 1,000 empirical variograms under the fitted models and used these to calculate the 95% point-wise tolerance intervals. In each case, the empirical variogram obtained from the data fell within the tolerance envelope, providing no evidence of a lack of fit. Because the 95% tolerance envelopes were relatively wide and primarily informative about spatial structure, we complemented the variogram-based diagnostics with an additional assessment of predictive calibration using posterior predictive probability plots. The empirical cumulative distribution functions of the envenoming and mortality models closely followed the expected linear relationship under a Uniform(0,1) distribution, indicating satisfactory predictive calibration. Therefore, considering the variogram-based diagnostics and predictive calibration assessment, we concluded that the fitted geostatistical models are compatible with the observed data.

#### Predictive mapping:

According to our modelling framework (lines 2–4 in Tables 2 and 4), incidence estimates vary systematically by the type of data-source. Community-based surveys yielded the highest incidence estimates, followed by hospital-based data and regional-level aggregated data, with the lowest estimates from country-level aggregated data. Country- and regional-level aggregated data produced similar estimates for both envenomings and deaths, while hospital and community-level data yielded comparable estimates for mortality. These systematic differences across data sources reflect heterogeneity in reporting intensity, case ascertainment, and aggregation, with country-level estimates likely arising from low-incidence settings, and hospital- and community-survey-based estimates likely arising from high-incidence settings.

We therefore generated *low*- and *high*-burden estimates for each country under different assumptions about plausible incidence and extent of data under-reporting. For high-income countries (HICs), we assumed that snakebite incidence is generally low and that under-reporting is relatively limited, given more complete reporting systems and higher health-seeking behaviour. Accordingly, country-level estimates were taken to represent the lowest plausible burden for all HICs, and no additional correction for under-reporting was applied; the same estimate is therefore reported for both the *low* and the *high* estimates. For all other countries, where the incidence is typically higher, and under-reporting is more likely due to limited surveillance infrastructure and lower health‑seeking behaviour, we defined the *low* estimate using hospital‑level predictions, intended to partially correct for under‑reporting while avoiding overestimation, and the *high* estimate using community-based survey predictions, intended to capture the full extent of under‑reporting in these settings. These *low* and *high* estimates represent alternative scenario assumptions and are not intended to represent statistical uncertainty bounds (e.g., confidence or predictive intervals) for a given region or country.

Our fitted models used country-wide average values of the explanatory variables and generated country-wide predictions as follows. Firstly, plugin predictions were made for each country by drawing 1,000 samples from the posterior predictive distribution for each country. Next, the average of these 1,000 samples was used as our point prediction. Finally, the range from the 25th to the 975th of the 1,000 ordered values defined the 95% predictive interval.

The geographical extent of categories one and two snakes was used to identify the population at risk, *n*, of envenomings and deaths in each country. This was achieved by overlaying the snake richness raster file on top of the population raster files. The same population raster file was used to calculate the total population, *N*, for each country. Point predictions of individual-level risk, *r*, were multiplied by the size of the population at risk, *n*, to obtain the predicted numbers, *rn*, of annual envenomings and deaths for each country. Annual incidence rates per 100,000 at risk were calculated as *I* = 100,000*rn/N*. Note that *n* = *N* when the snake distribution is not geographically restricted for a given country (i.e., I* = 100,000*r*). These predicted numbers are subject to a population-size effect; therefore, both *I* and *n* were reported to facilitate comparisons of risk and burden across countries. Predicted numbers and incidence rates were derived at the country level and did not account for within-country spatial variation in snakebite risk or population distribution.

Technical details of the analysis are provided in the S3 appendix. All analyses were performed in the R programming language version 4.5.1 [120]. Geostatistical modelling, including Monte Carlo maximum likelihood estimation were implemented using the PrevMap package version 1.2.2 [121].

## Results

### Snake envenomings

**Fig 1:**
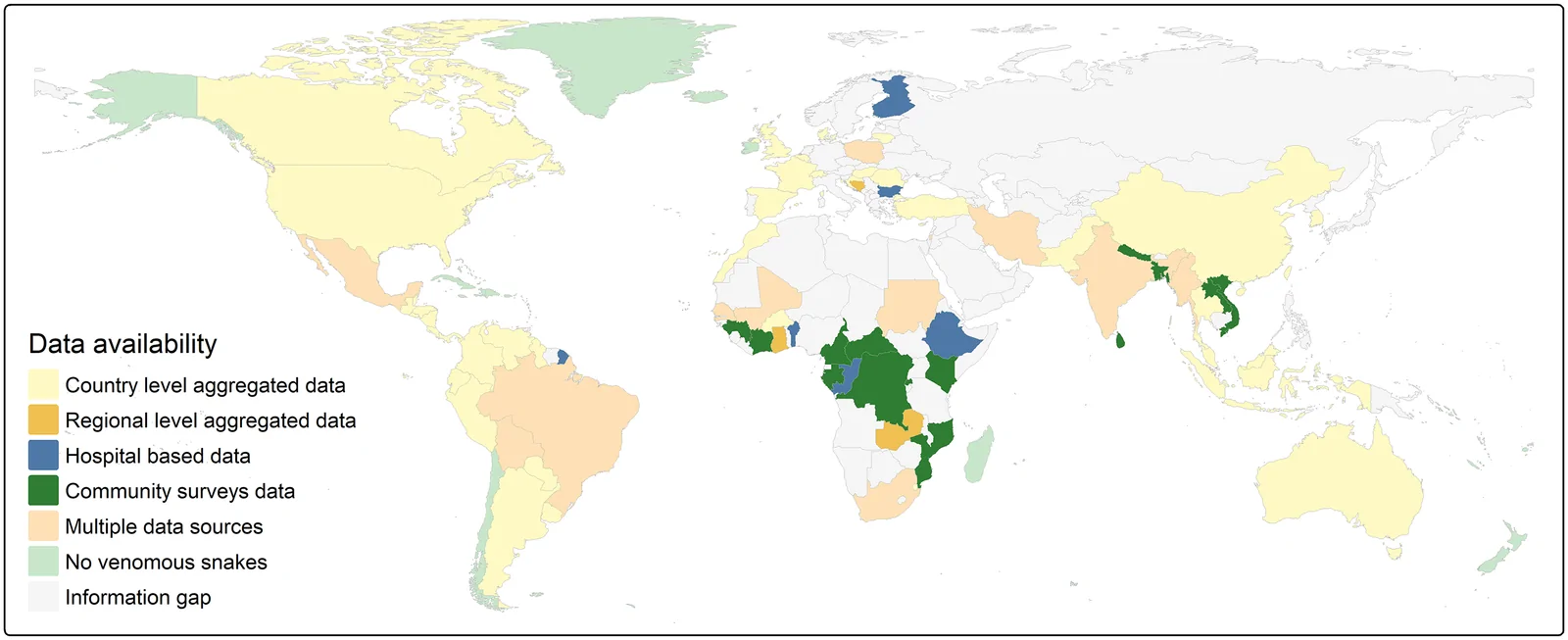
Global landscape of snake envenoming information availability. Base map adapted from spatial data provided by Natural Earth (https://www.naturalearthdata.com/downloads/50m-cultural-vectors/). The content on this site is licensed under a Creative Commons Attribution 4.0 International license (https://www.naturalearthdata.com/about/terms-of-use/).

Our analysis considered envenoming data from 79 countries. Among these, 42 (53.2%) had only country-level data, 3 (3.8%) had only regional-level data, 7 (8.8%) had only hospital-based data, 14 (17.7%) had community survey data, and 13 (16.5%) had two or more of the above (i.e., multiple sources). Fig 1 shows the distribution of data availability.

The geostatistical Poisson model identified the data source type, standard deviation (SD) of minimum temperature within a given year, percentage of urban population in a country (%) and Gender Inequality Index (GII) as explanatory variables for envenoming bites. Country-level, regional-level, and hospital-based data reported lower envenoming bites than community survey data. A lower incidence of envenoming bites was observed in countries with higher variation in minimum temperature and higher urban population. Higher envenoming bites were observed in countries with higher gender inequality. The envenoming data did not show temporal trends over the study period. The estimated variance of the Gaussian process (*σ^2^*) was 0.8, the nugget effect (*τ^2^*) was 1.0, and the scale parameter (*φ*) was 97 kilometres, indicating envenoming bites were spatially correlated up to approximately 300 km radius at a global scale (Table 2). Sensitivity analysis excluding imputed covariate values yielded consistent results; all covariates retained the same direction of associations, and most coefficient estimates differed by less than 10% from those of the primary analysis. The largest difference was observed for the hospital-source coefficient, which increased in absolute magnitude by 24.4%. Overall, exclusion of countries with imputed covariate values had minimal impact on model estimates and did not alter the interpretation of the findings (S3 Appendix).

**Table 2:** Geostatistical model for envenoming bites.

Variable	Estimate	Standard error	Z value	*P* value*
Intercept	−7.732	0.657	−11.760	<0.001
Source type (community survey - reference)				
Country	−2.192	0.337	−6.513	<0.001
Regional	−2.168	0.295	−7.344	<0.001
Hospital	−1.215	0.432	−2.810	0.005
Minimum temperature (SD)	−0.201	0.039	−5.072	0.006
Urban population (%)	−0.016	0.006	−2.718	<0.001
Gender Inequality Index	4.532	0.893	5.076	<0.001
Covariance parameters Matern function (kappa = 0.5)				
*σ^2^*	0.809	0.409		
*φ*	97.051	0.007		
*τ^2^/ σ^2^*	1.033	0.905		

*Parameter estimates, standard errors, and corresponding p-values were derived from a model-based geostatistical model. P values for the fixed effects were determined using two-sided Wald z-tests based on the ratio of the estimate to its standard error (z value).

**Fig 2:**
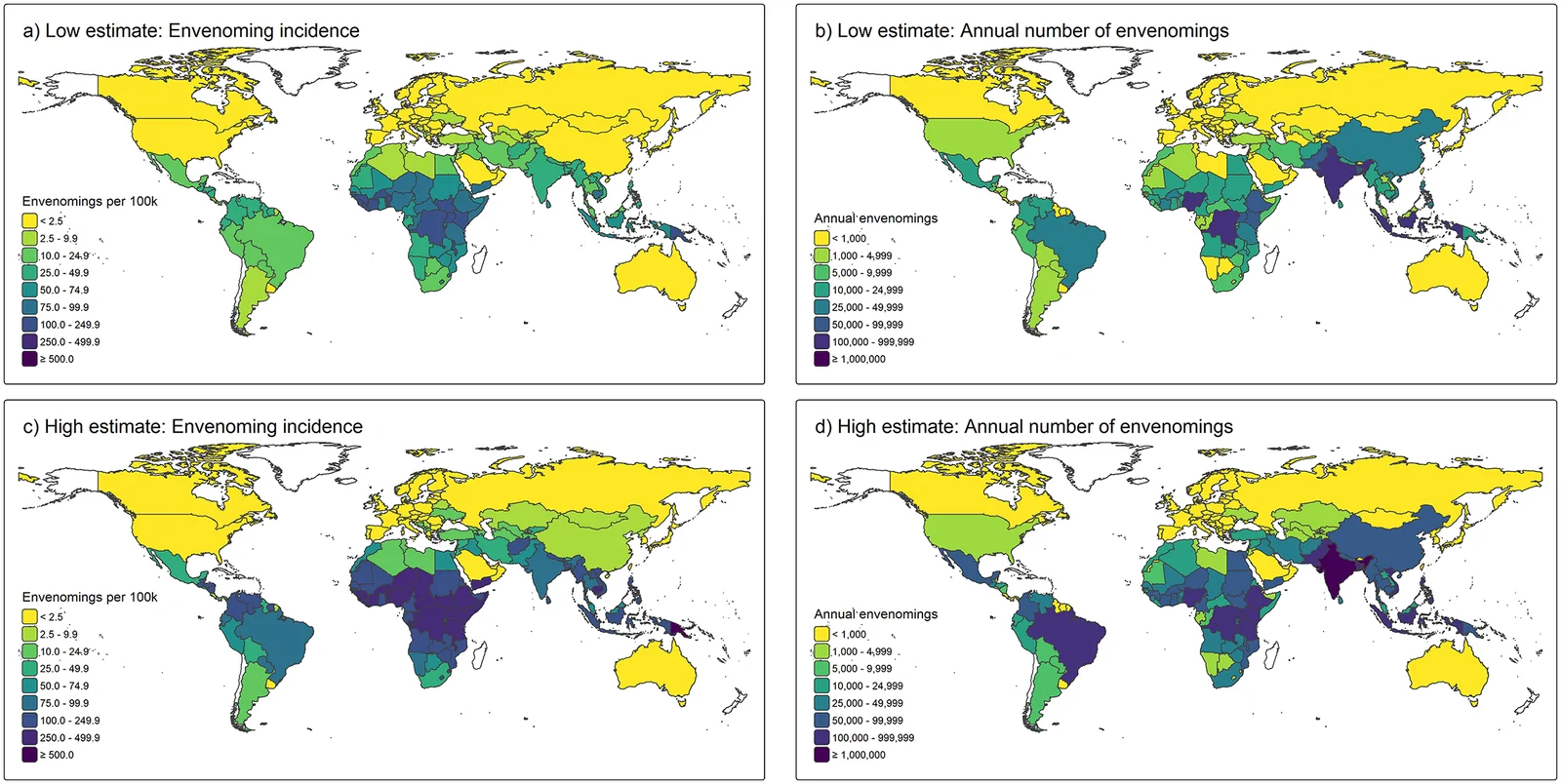
Global burden of envenomings: a) *low* estimate of envenomings incidence per 100,000 people. b) *low* estimate of annual envenomings. c) *high* estimate of envenomings incidence per 100,000 people. d) *high* estimate of annual envenomings. Countries without venomous snakes are shown wihtout shading. Base map adapted from spatial data provided by Natural Earth (https://www.naturalearthdata.com/downloads/50m-cultural-vectors/). The content on this site is licensed under a Creative Commons Attribution 4.0 International license (https://www.naturalearthdata.com/about/terms-of-use/).

Figure 2 presents *low* and *high* estimates of the global incidence of envenoming bites per 100,000 population and the corresponding annual number of bites. Table 3 summarises *low* and *high* estimates of the annual number of envenomings and corresponding incidence rates per 100,000 population, aggregated by global regions, World Bank income groups, and the 20 highest-burden countries. These 20 countries account for approximately 75% of global envenomings. India has the highest burden. Nigeria, Indonesia, and the Democratic Republic of the Congo are among the countries with very high burdens (see Table A in S4 Appendix for country-level estimates). The highest number of envenoming bites occurs in countries along the tropical belt, where temperature variability is minimal, creating ecological conditions that favour snake species.

**Table 3:** Estimated number of annual envenomings and incidence per 100,000 people (95% Predictive Interval) aggregated by global total, World Bank regions, income classifications, data availability, and top 20 countries with the highest total burdens (i.e., these top 20 countries collectively account for approximately 75% of the global burden).

	Low estimate (partial correction for under‑reporting)		High estimate (extensive correction for under‑reporting)	
**Annual envenomings**	**Incidence/100,000**	**Annual envenomings**	**Incidence/100,000**
**Global**				
Worldwide	2,100,000 [2,053,000, 2,149,000]	27.3 [26.6, 27.9]	7,047,000 [6,879,000, 7,226,000]	92.8 [90.6, 94.9]
**World Bank Regions**				
Sub-Saharan Africa	971,000 [944,000, 998,000]	86.3 [83.9, 88.7]	3,271,000 [3,180,000, 3,365,000]	290.8 [282.6, 299.1]
South Asia	567,000 [535,000, 599,000]	30.1 [28.5,31.9]	1,909,000 [1,808,000, 2,027,000]	101.5 [96.1, 107.8]
East Asia and Pacific	337,000 [316,000, 363,000]	14.5 [13.6, 15.5]	1,134,000 [1,062,000, 1,218,000]	48.6 [45.5, 52.2]
Latin America and Caribbean	131,000 [127,000, 136,000]	21.3 [20.6, 22.0]	440,000 [426,000, 455,000]	71.4 [69.1, 73.9]
Middle East and North Africa	74,600 [70,700, 79,000]	16.1 [15.3, 17.1]	250,000 [236,000, 265,000]	54.0 [50.9, 57.3]
Europe and Central Asia	15,700 [15,100, 16,400	1.7 [1.6,1.8]	39,300 [37,600, 41,300]	4.3 [4.1, 4.5]
North America	3,050 [2,950, 3,150]	0.8 [0.8–0.8]	3,050 [2,950, 3,150]	0.8 [0.8–0.8]
**Income group**				
Lower middle income	1,181,000 [1,141,000, 1,223,000]	40.6 [39.3, 42.1]	3,980,000 [3,844,000, 4,141,000]	136.9 [132.3, 142.5]
Low income	548,000 [531,000, 568,000]	86.6 [83.9, 89.8]	1,847,000 [1,789,000, 1,915,000]	292.0 [282.8, 302.8]
Upper middle income	358,000 [342,000, 376,000]	12.7 [12.2, 13.4]	1,208,000 [1,153,000, 12,700,00]	43.0 [41.0, 45.2]
High income	12,400 [11,900, 13,000]	0.9 [0.9, 1.0]	12,400 [11,900, 13,000]	0.9 [0.9, 1.0]
**Data availability**				
Countries that have data	1,486,000 [1,446,000, 1,527,000]	24.7 [24.0, 25.3]	4,989,000 [4,850,000, 5,131,000]	82.8 [80.5, 85.2]
Countries that do not have data	613,000 [588,000, 644,000]	36.5 [35.0, 38.4]	2,057,000 [1,971,000, 2,150,000]	122.6 [117.4, 128.1]
**Country**				
India	409,000 [384,000, 435,000]	29.3 [27.5, 31.2]	1,378,000 [1,290,000, 1,471,000]	98.7 [92.3, 105.3]
Nigeria	198,000 [181,000, 217,000]	92.0 [84.3, 100.9]	666,000 [610,000, 729,000]	309.8 [283.5, 339.0]
Indonesia	145,000 [130,000, 162,000]	53.7 [48.0, 59.9]	490,000 [438,000, 548,000]	180.9 [161.5, 202.1]
Democratic Republic of the Congo	139,000 [130,000, 147,000]	121.8 [114.2, 129.4]	467,000 [440,000, 495,000]	410.5 [387.2, 435.0]
Ethiopia	94,100 [86,400, 103,000]	90.7 [83.3, 98.9]	317,000 [292,000, 345,000]	305.7 [281.9, 332.7]
Kenya	66,300 [59,500, 74,800]	120.6 [108.4, 136.1]	224,000 [199,000, 252,000]	407.3 [362.6, 459.5]
Bangladesh	63,500 [48,300, 80,600]	39.4 [29.9, 50.0]	214,000 [166,000, 277,000]	133.0 [103.3, 172.0]
Uganda	53,200 [44,800, 62,800]	125.2 [105.4, 147.6]	179,000 [150,000, 213,000]	421.6 [353.5, 501.0]
Pakistan	51,100 [45,400, 57,500]	22.2 [19.7, 25.0]	171,000 [152,000, 192,000]	74.5 [66.3, 83.4]
Brazil	48,000 [46,400, 49,600]	22.9 [22.1, 23.7]	162,000 [156,000, 168,000]	77.1 [74.4, 80.0]
United Republic of Tanzania	43,400 [39,900, 47,200]	77.7 [71.3, 84.6]	146,000 [134,000, 159,000]	262.0 [240.4, 284.2]
Philippines	42,600 [31,000, 59,100]	43.3 [31.5, 60.1]	143,000 [103,000, 201,000]	145.8 [104.6, 204.8]
Vietnam	33,500 [26,300, 42,100]	34.7 [27.3, 43.7]	113,000 [88,200, 144,000]	117.1 [91.6, 149.5]
China	28,900 [27,900, 29,900]	2.0 [2.0, 2.1]	97,400 [94,100, 100,000]	6.9 [6.6, 7.1]
Ivory Coast	26,900 [23,200, 31,600]	103.9 [89.8, 122.3]	90,600 [78,400, 105,000]	350.3 [302.9, 406.0]
Burkina Faso	26,100 [21,900, 31,500]	114.7 [96.3, 138.4]	87,600 [73,100, 106,000]	384.5 [321.0, 463.2]
Yemen	24,900 [21,300, 28,700]	83.5 [71.5, 96.3]	84,200 [73,100, 98,200]	282.5 [245.0, 329.2]
South Sudan	23,200 [20,800, 26,200]	138.8 [124.6, 156.7]	78,200 [69,800, 88,100]	468.3 [417.8, 527.6]
Malawi	23,200 [17,400, 31,300]	124.1 [93.1, 167.6]	78,200 [58,200, 103,000]	419.2 [312.1, 553.4]
Sudan	21,600 [20,200, 23,000]	50.7 [47.4, 53.9]	72,800 [68,300, 77,600]	170.8 [160.4, 182.1]

Using our *low* estimates, 2.1 million envenomings occur worldwide each year. The *high* estimate suggests this figure could be as high as 7 million. sub-Saharan Africa bears the highest annual burden of snake envenomings globally (46%), followed by South Asia (27%) and the East Asia and Pacific region (16%). The Latin America and Caribbean region showed a substantial burden (6%), and the Middle East and North Africa a moderate burden (4%). The lowest burden was in Europe and Central Asia, and North America (<1%). The highest burden was observed in Low- and Middle-Income Countries (LMICs) (56%), followed by Low- Income Countries (LICs) (26%). Upper-middle-income countries (UMICs) showed a moderate burden (17%), and HICs showed the lowest burden (<1%). The incidence rates were highest in LICs, followed by LMICs and UMICs, with the lowest incidence observed in HICs.

We identified 170 countries with venomous snakes. Ninety-one of these (53.5%) countries lacked robust empirical data, including 18/21 (85.7%) countries in the Middle East and North Africa, 35/53 (66.0%) in Europe and Central Asia, 24/44 (54.5%) in sub-Saharan Africa, 9/21 (42.9%) in East Asia and Pacific, 2/7 (28.6%) in South Asia, and 3/22 (13.6%) in Latin America and Caribbean. The overall *low* annual estimate comprises 1.49 million (71%) envenomings in countries with available data and 613,000 (29%) in those without. Countries lacking data contribute to regional totals: 77.9% to the Middle East and North Africa, 50.6% to Europe and Central Asia, 46.4% to sub-Saharan Africa, 24.5% to East Asia and Pacific, 2.3% to South Asia, and 0.4% to Latin America and Caribbean. In our *high* annual estimates, the envenoming burden rises to 5 million in countries with data and to 2 million in countries without data.

### Snakebite mortality

**Fig 3:**
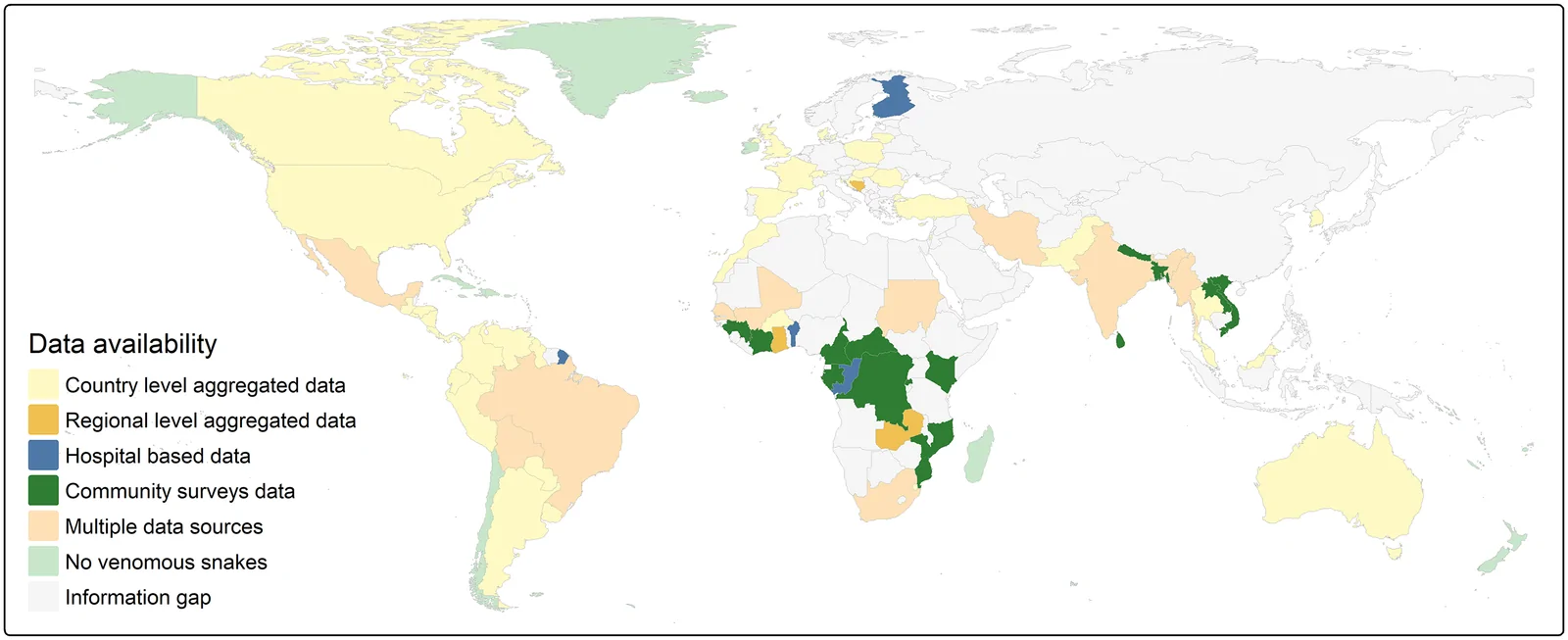
Global landscape of snake bite mortality information availability. Base map adapted from spatial data provided by Natural Earth (https://www.naturalearthdata.com/downloads/50m-cultural-vectors/). The content on this site is licensed under a Creative Commons Attribution 4.0 International license (https://www.naturalearthdata.com/about/terms-of-use/).

The snakebite mortality analysis included data from 70 countries. Thirty-eight of these (54.3%) had only country-level data, 3 (4.2%) had only regional-level data, 5 (7.1%) had only hospital-based data, 14 (20.0%) had community survey data, and 10 (14.3%) had two or more of the above (i.e., multiple sources). Fig 3 shows the distribution of data availability.

The geostatistical Poisson model identified the data source type, the maximum species richness of Category 1 snake species in a given area, carbon dioxide emissions per capita (production), and the Gender Inequality Index as explanatory variables for snakebite mortality. Country-level and regional-level sources reported lower snakebite mortality than community survey data. There was no difference in reported deaths between hospital-based data and community-based surveys. Higher mortality rates were observed in countries hosting more Category 1 snake species and those with greater gender inequality. A lower mortality was observed in countries with higher carbon dioxide emissions per capita (production). No temporal trends in envenoming-related deaths were detected over the study period. The estimated variance of the Gaussian process (*σ^2^*) was 2.3, the nugget effect (*τ^2^*) was 0.9, and the scale parameter (*φ*) was 339 kilometres, indicating that deaths were spatially correlated up to approximately 1,000 km radius at a global scale (Table 4). Sensitivity analysis excluding imputed covariate values yielded consistent results; all covariates retained the same direction of associations, and all coefficient estimates differed by less than 3% from those of the primary analysis, indicating that the imputation procedure had minimal influence on the model estimation (S3 Appendix).

**Table 4:** Fitted Geostatistical Poisson model for deaths.

Variable	Estimate	Standard error	Z value	*P* value*
Intercept	−13.503	1.135	−11.896	<0.001
Source type (community survey - reference)				
Country	−3.424	0.529	−6.473	<0.001
Regional	−3.373	0.485	−6.959	<0.001
Hospital	−0.626	0.685	−0.914	0.360
Category 1 snake species (maximum richness)	0.287	0.132	2.168	0.030
Carbon dioxide emissions per capita (production)	−0.177	0.088	−2.021	0.043
Gender Inequality Index	4.370	2.133	2.048	0.040
Covariance parameters Matern function (kappa = 0.5)				
*σ^2^*	2.299	0.118		
*φ*	338.942	0.001		
*τ^2^/ σ^2^*	0.962	0.716		

*Parameter estimates, standard errors, and corresponding p-values were derived from a model-based geostatistical model. P values for the fixed effects were determined using two-sided Wald z-tests based on the ratio of the estimate to its standard error (z value).

**Fig 4:**
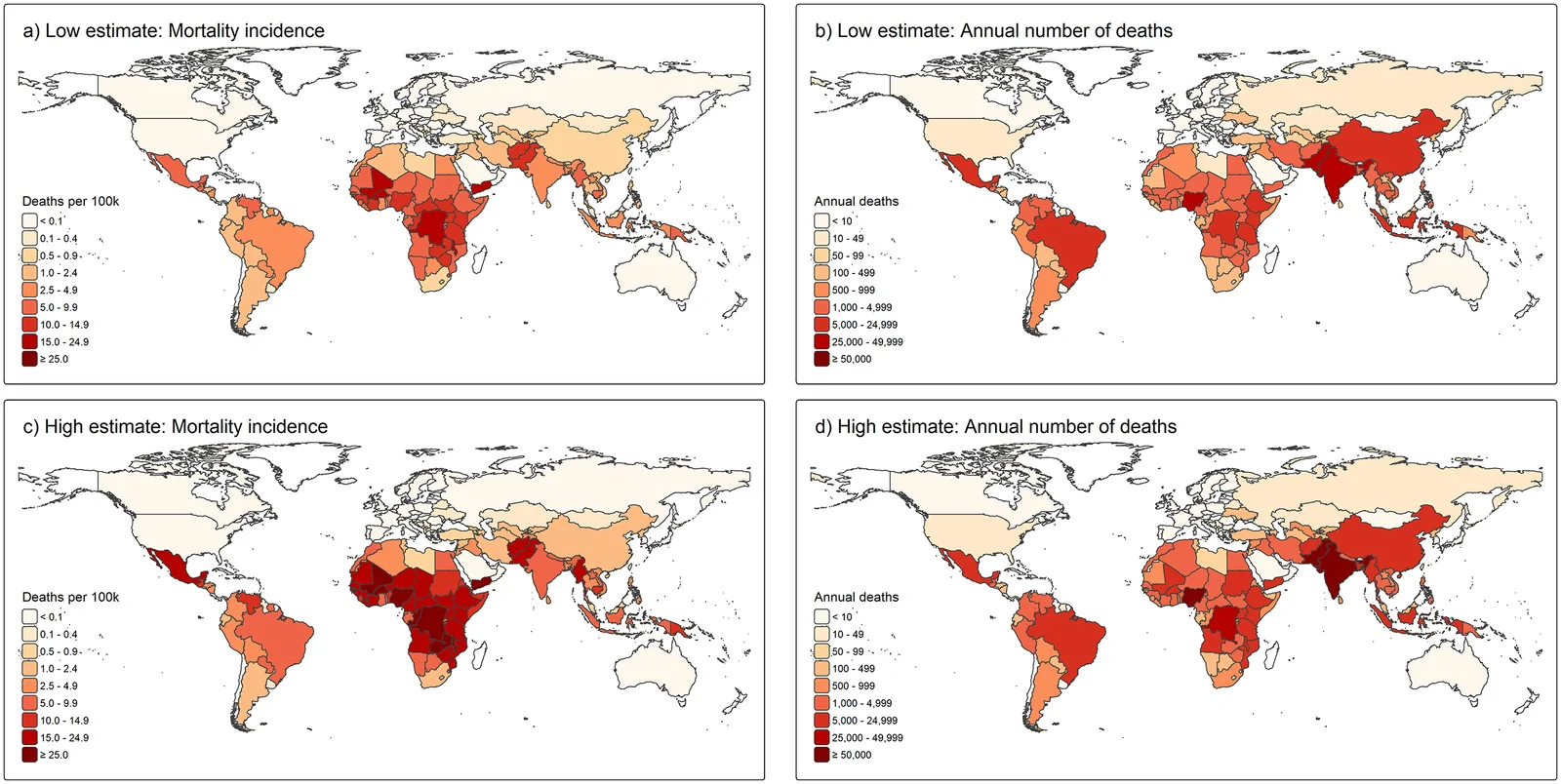
Global burden of deaths due to snakebites: a) *low* estimate of mortality incidence rate per 100,000 people per year. b) *low* estimate of the annual number of deaths. c) *high* estimate of mortality incidence rate per 100,000 people per year. d) *high* estimate of the annual number of deaths. Countries without venomous snakes are shown without shading. Base map adapted from spatial data provided by Natural Earth (https://www.naturalearthdata.com/downloads/50m-cultural-vectors/). The content on this site is licensed under a Creative Commons Attribution 4.0 International license (https://www.naturalearthdata.com/about/terms-of-use/).

Figure 4 shows *low* and *high* estmates for the global mortality incidence per 100,000 population and the corresponding annual number of deaths. Table 5 summarises *low* and *high* burden estimates of the annual deaths and the corresponding incidence rates per 100,000 population, aggregated by global, World Bank regions, income groups, and the 20 countries with the highest burden. These 20 countries share ~78% of global mortality. India bears the highest burden, and Nigeria, Pakistan, and the Democratic Republic of the Congo are among the countries with very high burdens (see Table B in S4 Appendix for country-level estimates).

**Table 5:** Estimated number of annual deaths and incidence per 100,000 people (95% Predictive Interval) aggregated by global total, World Bank regions, income classifications, data availability, and top 20 countries with the highest total burdens (i.e., these top 20 countries collectively account for approximately 78% of the global burden).

	Low estimate [partial correction for under‑reporting]		High estimate [extensive correction for under‑reporting]	
	Annual deaths	Incidence/100,000	Annual deaths	Incidence/100,000
**Global**				
Worldwide	274,000 [252,000, 296,000]	3.6 [3.3, 3.8]	513,000 [473,000, 559,000]	6.7 [6.1, 7.3]
**World Bank Regions**				
Sub-Saharan Africa	124,000 [108,000, 142,000]	11.0 [9.6, 12.6]	232,000 [203,000, 264,000]	20.6 [18.0, 23.5]
South Asia	74,800 [64,800, 868,00]	4.0 [3.4, 4.6]	140,000 [121,000, 164,000]	7.5 [6.4, 8.7]
East Asia and Pacific	34,500 [29,500, 40,800]	1.5 [1.3, 1.7]	64,400 [55,300, 76,900]	2.8 [2.4, 3.3]
Latin America and Caribbean	27,800 [25,000, 31,300]	4.5 [4.1, 5.1]	51,900 [46,700, 58,600]	8.4 [7.6, 9.5]
Middle East and North Africa	11,800 [10,000, 14,100]	2.5 [2.2, 3.0]	21,900 [18,500, 26,600]	4.7 [4.0, 5.7]
Europe and Central Asia	1,150 [950, 1,400]	0.1 [0.1, 0.2]	2,100 [1,750, 2,650]	0.2 [0.2, 0.3]
North America	35 [30, 35]	0.0 [0.0, 0.1]	35 [30, 35]	0.0 [0.0, 0.1]
**Income group**				
Lower middle income	148,000 [132,000, 165,000]	5.1 [4.5, 5.7]	277,000 [249,000, 312,000]	9.5 [8.6, 10.7]
Low income	71,200 [63,100, 80,500]	11.3 [10.0,12.7]	133,000 [117,000, 152,000]	21.1 [18.5,24.0]
Upper middle income	54,600 [49,900, 60,600]	1.9 [1.8,2.2]	102,000 [93,400, 113,000]	3.6 [3.3, 4.0]
High income	150 [100, 150]	0.0 [0.0, 0.1]	150 [100, 150]	0.0 [0.0, 0.1]
**Data availability**				
Countries that have data	198,000 [182,000, 214,000]	3.3 [3.0, 3.6]	370,000 [341,000, 403,000]	6.1 [5.7, 6.7]
Countries that do not have data	76,300 [66,200, 88,100]	4.5 [3.9, 5.2]	143,000 [124,000, 164,000]	8.5 [7.4, 9.8]
**Country**				
India	37,600 [32,000, 44,200]	2.7 [2.3, 3.2]	70,100 [58,800, 82,500]	5.0 [4.2, 5.9]
Nigeria	29,600 [22,700, 39,300]	13.8 [10.5, 18.3]	55,300 [42,400, 71,500]	25.7 [19.7, 33.2]
Pakistan	26,700 [20,600, 36,000]	11.6 [9.0, 15.6]	50,500 [38,200, 67,100]	22.0 [16.6, 29.2]
Democratic Republic of the Congo	20,700 [16,800, 25,300]	18.2 [14.8, 22.2]	38,700 [31,600, 46,600]	34.0 [27.8, 41.0]
Mexico	12,000 [9,800, 15,200]	8.6 [7.0, 10.9]	22,500 [17,900, 28,200]	16.1 [12.8, 20.2]
Indonesia	11,900 [8,550, 16,400]	4.4 [3.1, 6.1]	22,100 [16,600, 31,100]	8.1 [6.1, 11.5]
China	10,200 [9,200, 11,300]	0.7 [0.6, 0.8]	19,000 [17,200, 21,000]	1.3 [1.2, 1.5]
Ethiopia	9,400 [7,200, 12,100]	9.1 [6.9, 11.7]	17,600 [13,400, 23,200]	17.0 [12.9, 22.4]
Brazil	8,000 [7,250, 8,900]	3.8 [3.5, 4.2]	15,000 [13,500, 16,800]	7.2 [6.4, 8.0]
Kenya	7,200 [3,500, 12,600]	13.1 [6.4, 22.9]	13,500 [6,650, 24,000]	24.6 [12.1, 43.6]
United Republic of Tanzania	5,850 [4,100, 8,100]	10.5 [7.3, 14.5]	11,000 [7,550, 15,500]	19.7 [13.5, 27.8]
Yemen	4,900 [3,400, 7,200]	16.5 [11.5, 24.1]	9,100 [6,400, 13,500]	30.5 [21.5, 45.3]
Uganda	4,850 [2,350, 8,750]	11.4 [5.5, 20.5]	9,100 [4,400, 16,600]	21.4 [10.4, 39.0]
Myanmar	4,600 [3,050, 6,950]	9.7 [6.4, 14.7]	8,650 [5,650, 12,800]	18.2 [11.9, 26.9]
Mali	4,350 [3,150, 6,000]	18.7 [13.5, 25.8]	8,100 [6,000, 11,300]	34.8 [25.6, 48.6]
Afghanistan	4,000 [3,050, 5,550]	13.2 [10.0, 18.3]	7,550 [5,650, 10,500]	24.9 [18.7, 34.7]
Bangladesh	4,000 [1,900, 7,500]	2.5 [1.2, 4.7]	7,550 [3,550, 13,800]	4.7 [2.2, 8.6]
Burkina Faso	3,700 [2,150, 6,000]	16.2 [9.3, 26.3]	6,950 [4,150, 11,300]	30.5 [18.2, 49.6]
Angola	3,500 [2,750, 4,500]	9.9 [7.8, 12.8]	6,550 [5,150, 8,450]	18.5 [14.6, 24.0]
Sudan	2,950 [2,500, 3,450]	6.9 [5.9, 8.2]	5,500 [4,700, 6,550]	12.9 [11.0, 15.3]

Based on *low* estimates, snake envenomings cause over 274,000 deaths globally each year. The *high* estimates suggest this could be as high as 513,000. Sub-Saharan Africa records the highest annual mortality burden (45%), followed by South Asia (27%) and the East Asia and Pacific region (13%). Latin America and the Caribbean and the Middle East and North Africa experience substantial (10%) and moderate (4%) burdens, respectively. The lowest burden was in Europe and Central Asia, and North America (<1%). The burden is highest in LMICs (54%), followed by LICs (26%). UMICs exhibited an intermediate burden (20%), while HICs reported the lowest (<1%). The mortality rates were highest in LICs, followed by LMICs and UMICs, while HICs had negligible mortality rates.

Among the 170 countries with venomous snakes, 100 countries lacked robust empirical mortality data, including 37/53 (69.8%) countries in Europe and Central Asia, 18/21 (61.6%) in the Middle East and North Africa, 13/21 (61.9%) in East Asia and Pacific, 25/44 (56.8%) in sub-Saharan Africa, 2/7 (28.6%) in South Asia and 5/22 (22.7%) in Latin America and Caribbean. Our *low* estimate for countries with empirical data is 198,000 (72%) deaths annually, while for countries lacking data, it is 76,300 (28%). The countries lacking data contribute to the regional total as follows: 70.3% to Europe and Central Asia, 82.6% to the Middle East and North Africa, 74.2% to East Asia and Pacific, 54.8% to sub-Saharan Africa, 0.5% to South Asia, and 0.2% to Latin America and Caribbean. According to our *high* estimates, the death toll in countries with available data rises to 370,000, while in countries lacking data it increases to 143,000.

## Discussion

Based on our analysis, we estimate that at least 2.1 million snake envenomings occur annually, resulting in 274,000 deaths worldwide. However, these figures likely represent a conservative baseline; the true burden may be substantially higher, potentially up to three times greater for envenomings and twice as high for mortality. The highest burden of snake envenoming is concentrated among countries within the tropical belt, particularly in sub-Saharan Africa and South and Southeast Asia. Although LMICs report the highest number of envenomings, the highest envenoming incidence is observed in LICs, highlighting the severity of the burden that further strains healthcare systems in the most resource-poor settings. Interestingly, sub-Saharan Africa accounts for 45% of global snake envenomings and deaths, with nearly three times the incidence rates compared to South Asia, a pattern not previously identified [122]. LICs exhibit the highest mortality rates, which can be attributed to the compounded effects of economic constraints, weak healthcare infrastructure, and limited access to antivenom [107]. Therefore, WHO’s 2030 target of reducing mortality and morbidity due to snake envenoming by 50% presents a formidable challenge, particularly in high-burden regions [123].

These revised estimates substantially exceed our previous estimates and those currently quoted by WHO [124]. We identified 170 countries with venomous snakes; however, 91 (53.5%) countries lacked robust data on envenomings, including 86% of countries in the Middle East and North Africa, 55% of countries in sub-Saharan Africa, and 43% of countries in the East Asia and the Pacific region. Notably, 30% of our estimate of the global burden of envenomings was attributed to countries that lacked empirical data. Furthermore, 100 (58.8%) countries lacked mortality data, and 39% of the global mortality burden was attributed to countries that lacked empirical data. This highlights not only the need for model-based estimates if we are to assess the true global burden, given the lack of empirical data, but also the urgent need for more countries to undertake burden assessments, even at a basic level, to improve data completeness and reliability.

Previous global estimates of snakebite burden were largely derived by applying incidence rates to national and regional populations, often based on extrapolations from limited regional studies. Notably, Kasturiratne and colleagues acknowledge that this approach likely underestimates the true burden, due to sparse data and reliance on assumptions [16]. A few other available estimates lacked transparent methodologies and were not reproducible, limiting their utility for policy planning [15,125]. In this study, we adopted geostatistical modelling techniques implemented in open-source software, using publicly available social and natural environmental covariate data to estimate global snake envenomings and mortality. Recognising that snakebite disproportionately affects impoverished rural populations in tropical regions [18,126], we selected explanatory variables that best capture the socioeconomic, climatic and environmental determinants of snakebite risk [108,127–129]. This multidimensional approach ensured that the model accounts for both human vulnerability and snake ecology, thereby enhancing the predictive capacity and burden estimates across diverse geographic contexts. The inclusion of these explanatory variables enables the prediction of outcomes for countries lacking empirical data, thereby enhancing the generalizability of our approach [13,130]. Datasets derived from hospital-based sources are subjected to substantial ICD-10 coding inconsistencies. Recently, linked-data analysis demonstrated that toxicology-related codes can misclassify envenoming presentations, potentially inflating case counts and deaths [131]. To account for these systematic differences, we included the data source as a covariate and used a Gaussian process to capture unmeasured variability arising from miscoding and reporting bias. Unlike previous estimates, we considered multiple studies available for the same country with different data sources. Our methodology enabled us to provide unbiased estimates using multiple data points within a country and data from neighbouring sources and addresses the complexity of snakebite occurrences by capturing the interaction among humans, snakes, and the environment. This is achieved through a combination of explanatory variable adjustments and spatially correlated residual variation, which represent explained and unexplained spatial variation, respectively. This approach is applicable to other contexts in which global-scale mapping requires the pooling of information from multiple data sources, some of which are susceptible to under-reporting. Including climatic variables raises the possibility that global climate change may affect snakebite risk, as shifting temperature patterns affect both snake ecology and human exposure. This warrants further investigation into snakebite vulnerability under future climate scenarios, particularly in regions projected to experience ecological shifts [12,130,132,133].

Our primary objective was to estimate the global burden, and the individual country-level predictions are provided as supporting outputs to assist policymakers, particularly those in countries without robust empirical epidemiological data, in gaining preliminary insights into their likely burden. We do not expect our models to perform equally well across all settings, nor do we present country-level outputs as unbiased point estimates. Our estimates for low-risk high-income countries, where snakebite is rare, nonenvironmental exposure dominates, and good surveillance systems already exist, should not be interpreted in the same way as estimates for LIC and LMIC regions. We explicitly encourage cautious interpretation of country-level outputs, especially in settings where endemic snakebite risk is low or when high-quality local surveillance data already exist. In such contexts, national or sub-national empirical data should take precedence over global burden outputs. Conversely, in data-poor settings, our models provide a structured range of plausible burden scenarios that can inform priority-setting and motivate targeted epidemiological data collection.

Our analysis showed that 20 countries account for 75% of the global envenomings, highlighting the need to prioritise these nations for targeted prevention and management strategies. In our *high* estimates, 65 countries exhibit an overall incidence of over 100 envenomings per 100,000 people. Forty-six% of them had no published data available, and only around 28% of these high-burden countries had conducted community-based surveys, indicating a far greater need to identify snakebite as a national public health problem and take proactive steps to quantify the burden. We found that 20 countries accounted for 78% of all snakebite-related deaths. In our *high* estimates, 45 countries exhibited a mortality incidence of over 10 deaths per 100,000 people, with patterns similar to envenomings - some countries conducting detailed surveys, but many with no data.

While snakebite deaths are a subset of envenoming cases, some sources reporting envenomings did not include mortality data, and some mortality datasets lacked corresponding envenoming figures. Additionally, the ratio of envenoming to deaths is likely to vary substantially across regions due to differences in the composition of Category 1 and Category 2 snake species, as well as variations in healthcare access and treatment availability. Therefore, we developed separate models to estimate envenomings and deaths resulting from snakebites. Our analysis revealed systematic differences in incidences across different data sources. Community-based surveys consistently reported the highest incidence rates for envenomings, followed by hospital-based datasets, whereas country-and regional-level aggregated data yielded the lowest estimates. A similar pattern was observed for mortality in community-based, country- and regional-level aggregated data. However, hospital estimates were often higher than country and regional level aggregated data, and were not different to community surveys, suggesting that most victims of fatal bites reach hospitals at some point, or that most deaths occur in hospitals. This pattern may reflect two distinct phenomena: first, underreporting of numbers in hospital-based, country, and regional-level aggregated data in envenomings, and underreporting in country- and regional-level aggregated data in deaths; second, low incidences in certain countries. The former is likely to be found in LMICs and LICs, where robust data collection platforms are often absent and traditional health-seeking behaviours are prevalent. The latter is likely in HICs, with low numbers due to low incidence rates. Additionally, HICs have more robust reporting systems, and underreporting is less likely.

In our analysis, we produced two sets of estimates to gain a deeper understanding of the situation in individual countries. We adopted a scenario-based approach to address substantial heterogeneity in surveillance capacity, health-seeking behaviours, and data availability across countries. We generated *low* estimates applying country-level predictions for HICs and hospital-level predictions for the rest of the countries. We chose this option because it was more suitable for HICs with relatively low incidence rates, for other countries with generally higher incidence rates, and for countries lacking empirical data. This approach yielded intermediate predictions that strike a balance between conservatism and realism, acknowledging there to be some degree of underreporting while avoiding overestimation. We generated *high* estimates applying country-level predictions for HICs and survey-level estimates for the rest of the countries, which reflect the burden that would be expected if comprehensive community-based surveys were conducted. These *high* estimates aim to capture the extent of underreporting in settings with limited surveillance infrastructure and low levels of health-seeking behaviour. Together, this stratified methodology provided a more nuanced and context-sensitive representation of the global burden of snake envenomings.

Given that the primary objective of the modelling framework was to predict snakebite burden rather than estimate causal effects, covariates were prioritised according to their contribution to explaining spatial variation and improving predictive performance. Consequently, the associations described below should be interpreted as predictive rather than causal and are intended to support burden estimation rather than to infer causal conclusions about the determinants of snakebite risk. Explanatory variables of the model for envenoming included minimum temperature (SD), urban population (%), and GII. Envenoming incidence decreases when the variation of minimum temperature increases. Minimum temperature variation shapes the climatic and ecological dynamics of tropical and temperate regions. Furthermore, low variation in minimum temperatures sustains year-round snake activity, contributing to a persistent envenoming risk [134]. These findings underscore the need for global climate-informed public health strategies to mitigate snakebite risk [12]. Unsurprisingly, lower envenoming rates were observed in countries with a higher percentage of urban populations, suggesting that snakebites are a significant problem in rural areas [1]. Higher envenoming numbers are seen in countries with higher GII. Gender inequality is a determinant and consequence of poverty and underdevelopment in low-income countries (acting as a driver of poverty and a barrier to development). Gender inequality is a fundamental economic constraint that limits women’s access to education, employment, healthcare, and political participation, thereby reducing household income, productivity, and national economic growth. Therefore, countries with a higher GII tend to have higher poverty and lower human development, indicating snakebite is a disease of poverty [1].

Explanatory variables in the snakebite mortality model included the maximum Category 1 snake species richness in a given area, Carbon dioxide emissions per capita (production) and the GII. The relationship with WHO Category I snakes is not surprising, as these represent the most medically significant venomous species worldwide, accounting for the majority of snakebite-related mortality. Their venom profiles often result in systemic complications, including neurotoxicity, coagulopathy, and renal failure. Beyond clinical severity, these species present unique treatment challenges; their wide taxonomic diversity leads to uncertainty in diagnosis, variability in treatment efficacy, and difficulties in producing antivenom. Multispecies antivenoms are often required; however, they are complex and costly to manufacture, and their effectiveness can vary across regions. Combined with limited access to antivenom and inadequate healthcare infrastructure in tropical and subtropical regions, these factors amplify the risk of fatal outcomes [18]. The explanation for the independent relationships between carbon dioxide (CO_2_) emissions per capita (production) and the GII and snakebite mortality is most likely to be indirect. A negative relationship exists between national carbon dioxide emissions per capita and snakebite mortality, while a positive relationship is observed between the Gender Inequality Index and snakebite mortality, likely reflecting the broader socioeconomic and infrastructural disparities between high- and low-income countries. High CO_2_-emissions and low GII nations tend to have advanced healthcare systems, urbanised populations, and mechanised agriculture, all of which reduce snakebite risk and improve outcomes. In contrast, low-emission and high-GII countries often face high snakebite mortality due to their large rural communities, high ecological exposure to venomous snakes, inadequate access to antivenom, and under-resourced health systems [1].

The spatial structure of envenoming bites and associated mortality exhibits distinct patterns of spatial dependence. For envenoming bites, the estimated variance of the Gaussian process (0.8), the nugget effect (1.0), and the scale parameter (97 kilometres) suggest a relatively moderate spatial correlation structure with short-range spatial dependence, extending up to 300 km. This indicates that local environmental and ecological factors, as well as finer-scale patterns of venomous snake occurrence and habitat distribution, may be driving the clustering of envenoming bites. In contrast, the spatial model for deaths exhibits a higher estimated variance of the Gaussian process (2.3), a lower nugget effect (0.9), and a much larger scale parameter (339 kilometres), implying spatial correlation extending up to 1,000 kilometres. This indicates that mortality outcomes are influenced by broader regional determinants, potentially including systematic healthcare disparities, referral pathways, antivenom availability, and regional differences in snake species or venom toxicity. These findings have implications for targeting interventions, where prevention strategies such as community education and using protective footwear may need to be localised, while mortality reduction efforts should be coordinated at a broader regional or even national scale [135,136].

The global burden of snake envenoming remains underestimated, particularly in rural and underserved regions where victims often exhibit diverse health-seeking behaviours and the reporting systems remain fragmented or weak [137]. Based on an assumption that approximately 50% of bites result in envenoming, we estimate that at least 4 million snakebites occur globally each year; the true number may be three times higher [107]. Our estimates did not explore the chronic disability associated with snake envenoming. Nevertheless, assuming amputations and other permanent physical disabilities occur at approximately three times the number of deaths [4], we can estimate that at least 825,000 cases of severe physical disabilities occur annually. To this must be added the chronic psychological disability associated with snakebite [3]. Together, these demonstrate the considerable burden of long-term morbidity related to snake envenoming, leading to loss of productivity and other adverse socio-economic consequences, which remain largely underrepresented in global health metrics [138].

There are potential flaws in the data sources we used due to the lack of robust national data. Most published community surveys on snakebite have been conducted in areas within a country where snakebite is endemic and potentially a significant public health problem, which can sometimes lead to an overestimation of the nationwide burden. It is also challenging to determine whether some community surveys included patients with bites from nonvenomous snakes or dry bites from venomous snakes. When the study did not explicitly state whether all bites or envenoming bites were included, we assumed 50% of all bites were venomous. However, the proportion of envenomings among recorded bites is likely to have varied geographically, depending on snake species. Hospital data are also sometimes flawed in that they may miss people with mild envenoming who may not seek hospital treatment or some patients with moderate or severe envenoming who die before reaching the hospital or who simply cannot access hospital care.

In any estimation, the accuracy of the estimate depends on the accuracy of available data and the assumptions used. Fitting a single model assumes that the relationship between explanatory variables and snakebite outcomes remains broadly consistent across regions. The LICs and LMICs, which contribute most of the data, are therefore naturally dominant in the spatial signal. Although the geostatistical modelling framework accounted for residual heterogeneity through stochastic error components, including variability across data sources, it may not have fully captured systematic differences in case ascertainment between country-level, regional-level, hospital-based, and community-based data sources. Such differences may arise from variation in health-seeking behaviour, healthcare access, reporting practices, and surveillance intensity. Consequently, some residual bias may remain in estimates derived from heterogeneous data sources. We acknowledge that in countries with no empirical data, model predictions may be influenced by incidental patterns in neighbouring settings. This behaviour is inherent to spatial models that borrow strength across geographic areas where direct observations are unavailable. While such information burrowing is a major advantage of spatial modelling*,* it can also be a limitation when neighbouring countries differ substantially in ecological, demographic, and health system characteristics. Additional high-quality data would further improve model performance in these settings. Therefore, country-specific estimates in such data-sparse settings should be interpreted with caution. However, predictions for countries without empirical data are informed by both the fixed-effect (socioeconomic and environmental) explanatory variables and the residual spatial Gaussian process, thereby reducing inappropriate cross-regional borrowing of information and smoothing. Although spatial averaging and bilinear interpolation provided the most parsimonious and stable representation of the explanatory variables for our prediction-focused aims, we acknowledge that residual exposure misclassification may persist in countries with substantial subnational heterogeneity. Therefore, country-specific estimates in such data-sparse settings should be interpreted with caution.

Envenoming and mortality estimates are from two models that are not nested and share only a single explanatory variable, GII; consequently, envenoming and mortality estimates might not be concordant. Therefore, case fatality rates (CFR) must be carefully assessed and interpreted. The mortality model is based on less data and covers fewer geographical regions than envenoming bites. Additionally, mortality is a rarer outcome than envenomings, the mortality model is informed by fewer observed events, which reduces statistical power and results in lower prediction precision. This leads to wider predictive intervals, and this should be borne in mind when interpreting mortality data. The higher CFRs observed in hospital-based scenarios likely reflect the inherent severity bias of hospital data, which disproportionately captures severe envenomings and thus exhibits higher mortality rates. Although we adjust for underreporting in hospital-level estimates, some residual upward bias may persist. A small number of countries show high CFRs. These patterns reflect uncertainty in data-poor settings rather than the true lethality and should therefore be interpreted with caution.

Our modelling approach assumes that ecological and environmental factors drive snakebite risk. However, in some countries, particularly in HICs, a proportion of snakebites arises from nonenvironmental exposures, such as those occurring in zoos, herpertariums, private collections, or exotic pet ownership rather than from naturally occurring snake populations. The explanatory power of specific and environmental covariates is limited in these contexts, and our models may deviate from the endemic snakebite risk in such settings. Even under conservative assumptions that snakebite risk is overestimated across all HICs, their combined contribution to the global burden remains small, amounting to approximately 12,500 envenomings and 150 deaths in total. This represents only a minor fraction of the global burden (0.6% of envenomings and 0.05% of deaths), indicating the potential overestimation in high-income settings does not materially influence global conclusions.

We aimed to estimate the global burden and to provide estimates for countries lacking accurate data. The numbers of envenomings and deaths were calculated by multiplying the estimated incidence by the population at risk. Consequently, countries with large populations may show high numbers, even if the estimated incidence rate is very low. This reflects a population-size effect rather than elevated risk in countries. Accordingly, incidence rates provide a more appropriate basis for comparing risks across countries, whereas estimated numbers are more informative for understanding the scale of the burden and health-system needs. Further, these country-level predictions do not capture within-country heterogeneity in snakebite risk. In many settings, snakebite burden is highly localised and concentrated in specific ecological or occupational contexts, rather than being evenly distributed across entire countries. Further, population density did not appear to improve the predictive performance during exploratory analysis. Consequently, no explicit adjustment was made for population density in the estimation of snakebite risk, even though snakebite risk is generally low in highly populated areas. As a result, country-level summaries should not be interpreted as indicating homogenous national risk or uniform exposure.

Given the limited data and heterogeneity in data quality across countries, we are not able to provide a single definitive estimate for each setting. Instead, we present a structured range of estimates, allowing decision-makers to choose the scenario that best reflects their empirical knowledge, local surveillance capacity and health system context.

Seventeen years after our original estimate, it is even clearer than previously that snake envenoming causes significant morbidity and mortality globally, particularly in sub-Saharan Africa and South and Southeast Asia, and there is a desperate need for more robust snakebite epidemiological data. Unlike previous approaches that relied on aggregated incidence rates, our model accounts for spatial heterogeneity and ecological drivers of risk, producing country-level estimates and maps. These outputs can inform targeted interventions, optimise antivenom deployment, and guide future research on environmental and socio-demographic determinants of snakebite. Furthermore, our methodology may also be applicable for estimating the burden of similar diseases, especially in resource-limited settings where uniformly reliable data are not available.

## Supporting information

S1 Appendix Data. (CSV)

S2 Appendix Explanatory variables. (DOCX)

S3 Appendix Technical details of the analysis. **Table A:** Non-spatial model for snake envenoming incidence. **Table B:** Assessing multicollinearity among explanatory variables included in the non-spatial model for snake envenoming incidence. **Table C:** Non-spatial model for snakebite mortality incidence. **Table D:** Assessing multicollinearity among explanatory variables included in the non-spatial model for snakebite mortality incidence. **Table E:** Geostatistical model for envenoming bites after excluding countries with imputed covariates. **Table F:** Geostatistical Poisson model for deaths after excluding countries with imputed covariates. **Fig A:** Variogram of the point predictions of standardised Pearson residuals for A) envenoming bites and B) snakebite mortality. Variograms of the point predictions give evidence against the assumption of spatially uncorrelated standardised Pearson residuals for both envenoming bite incidence and snakebite mortality incidence. **Fig B:** Convergence diagnostics: (A) autocorrelogram and (B) trace plot of simulated samples for envenoming bite incidence; (C) autocorrelagram and (D) trace plot of simulated samples for snakebite mortality incidence. **Fig C:** Empirical log incidence versus explanatory variables of the model. **Fig D:** Empirical log mortality incidence versus explanatory variables. **Fig E:** Assessing the goodness of the fit of the geostatistical models for envenoming bite incidence and snakebite mortality incidence. The solid line is the empirical variogram of the data. The grey colour envelope indicates the 95% confidence bands under the fitted spatial model. The empirical variograms obtained from the envenoming data and mortality data fall within the respective envelopes of 95% tolerance limits. **Fig F:** Calibration assessment of the geostatistical model. The figures display the Posterior Predictive Calibration of the fitted spatial models for envenoming bites and snakebite mortality.
(DOCX)

S4 Appendix Supplementary Tables. **Table A:** Estimated envenoming incidence per 100,000 people and number of annual numbers of envenomings (95% Predictive Interval) derived from country‑level estimates (reflecting no correction for under‑reporting), derived from hospital‑based estimates (reflecting partial correction for under‑reporting), and derived from community‑based survey estimates (reflecting extensive correction for under‑reporting). Please note that no corrections have been made for high-income countries (HICs) in hospital-based and survey-based estimates. *Estimates presented in the manuscript as *low* estimate and *high* estimate scenarios. **Table B:** Estimated mortality incidence per 100,000 people and number of annual number of deaths (95% Predictive Interval) derived from country‑level estimates (reflecting no correction for under‑reporting), derived from hospital‑based estimates (reflecting partial correction for under‑reporting), and derived from community‑based survey estimates (reflecting extensive correction for under‑reporting). Please note that no corrections have been made for high-income countries (HICs) in hospital-based and survey-based estimates. *Estimates presented in the manuscript as *low* estimate and *high* estimate scenarios.
(DOCX)

## Abbreviations

CDC: Centers for Disease Control and Prevention
CFR: case fatality rates
GII: Gender Inequality Index
HICs: high-income countries
LICs: Low- Income Countries
LMICs: Low- and Middle-Income Countries
MCMC: Markov chain Monte Carlo
SD: standard deviation
SE: Snake envenoming
UMICs: Upper-middle-income countries.
