# Supplementary material for: Estimates of global burden of snakebite: A literature review and geostatistical modelling study
Source: PLoS Med. 2026 Aug 25;23(8):e1005201. doi: 10.1371/journal.pmed.1005201 (PMC13505955; doi:10.1371/journal.pmed.1005201)
Supplement: Supplementary file 1 [file pmed.1005201.s002.docx]

## Explanatory variables

We selected explanatory variables that capture the underlying natural and social environmental conditions associated with snakebite risk. Our explanatory variables included snake distribution, socioeconomic, demographic, climate, environmental, agriculture and farming-related variables. The following explanatory variables were considered.

Country-level United Nations Development Programme for the years 2005, 2010, 2015 and 2020 (<https://hdr.undp.org/data-center/documentation-and-downloads>)

- Health and Demographics -Life Expectancy at Birth for the overall population and by gender, Adolescent Birth Rate, Child mortality, Maternal Mortality Ratio, and Nutrition
- Education - Years of Schooling and Expected Years of Schooling for the overall population and by gender, School attendance, and percentage of the Population with at least some secondary education
- Economic and Social Status - Gross National Income Per Capita and Labour force participation rate for the overall population and by gender, Share of seats in parliament by gender, and Assets
- Inequality and Development - Human Development Index, Inequality-adjusted Human Development Index, Gender Development Index, and Gender Inequality Index, Coefficient of human inequality, Difference from HDI value, Inequality in education, Inequality in income, Inequality in life expectancy, and Overall loss
- Environmental and Sustainability - Carbon dioxide emissions per capita, Material footprint per capita, and the Planetary pressures adjusted Human Development Index
- Poverty -Multidimensional Poverty Index, access to Cooking fuel, Drinking water, Electricity, Sanitation, Housing, and Years of schooling

Country-level data on the share of land area used for arable agriculture for the years 2005, 2010, 2015 and 2020 were obtained from the Food and Agriculture Organization of the United Nations, via the World Bank (2025) (<https://ourworldindata.org/land-use>).

Country-level data on the share of the population living in urban areas for the years 2005, 2010, 2015 and 2020 were obtained from the United Nations Population Division, via World Bank (2025) (<https://ourworldindata.org/grapher/share-of-population-urban?time=2023>)

Spatially continuous surfaces of monthly minimum temperature, maximum temperature, and precipitation with 5 km x5 km resolution from 2000 to 2024 were obtained from WorldClim (<https://www.worldclim.org/data/monthlywth.html>).

Spatially continuous surface of elevation with 5 km x 5 km resolution was obtained through the elevatr package in R (Hollister, J., Shah, T., Nowosad, J., Robitaille, A., Beck, M., & Johnson, M., 2023). elevatr: Access Elevation Data from Various APIs. R package version (Hollister J, Shah T, Nowosad J, Robitaille AL, Beck MW, Johnson M. elevatr: Access Elevation Data from Various APIs. 2025. doi:10.5281/zenodo.8335450).

Spatially continuous surfaces of category one and two venomous and medically important snake species distribution, with 5 km x 5 km resolution, were obtained from a previous publication (Longbottom J, Shearer FM, Devine M, Alcoba G, Chappuis F, Weiss DJ, et al. Vulnerability to snakebite envenoming: a global mapping of hotspots. The Lancet. 2018;392: 673–684. doi:10.1016/S0140-6736(18)31224-8).

Population counts data (i.e. Unconstrained global mosaic with 1 km resolution) for 2005, 2010, 2015 and 2020 were obtained from WorldPop. Population counts, aggregated to 5km x 5 km resolution, were used for the analysis. (<https://hub.worldpop.org/geodata/listing?id=64>).
