# Supplementary material for: Estimates of global burden of snakebite: A literature review and geostatistical modelling study
Source: PLoS Med. 2026 Aug 25;23(8):e1005201. doi: 10.1371/journal.pmed.1005201 (PMC13505955; doi:10.1371/journal.pmed.1005201)
Supplement: Supplementary file 2 [file pmed.1005201.s003.docx]

# Exploratory analysis - Generalised linear modelling of envenoming bite and snakebite mortality

The data sources provided the number of envenomings and deaths across different geographical areas. This included country-level aggregated data, regional-level aggregated data, and data from hospital catchment areas and surveys.

Categorical explanatory variables were included directly in the modelling, as they do not vary within countries. When an explanatory categorical variable was missing for a given country, we imputed its value using the average of neighbouring countries, assuming their regional similarity. Missing values were limited to six countries for envenomings (Central African Republic, French Guiana, Hong Kong, Martinique, Occupied Palestinian Territory, and Taiwan) and four for mortality (Central African Republic, Hong Kong, Occupied Palestinian Territory, and Taiwan). We adopted this approach to avoid excluding countries from the analysis, given the widespread data scarcity in SBE.

The quantitative (continuous) explanatory variables show spatial heterogeneity within geographical regions. Therefore, we evaluated multiple extraction approaches to identify the representation that provided the strongest predictive performance. For country-level and regional-level studies, the epidemiological outcome data were reported as counts aggregated by administrative units (e.g. countries or regions). This aggregation masks the underlying spatial variation within the outcome data. We therefore extracted mean values of explanatory variables across the extent of each administrative unit. This approach ensured that the explanatory variables were spatially aligned with the resolution of the outcome data, providing the most representative spatial summary for these data sources. For community surveys and hospital data, we extracted explanatory variable values at the centroid of each study location and calculated spatially averaged values across multiple radii (10 km, 20 km, 50 km, 100 km, 250 km, 500 km, and 1000 km). This approach allowed us to explore the spatial scale at which explanatory variables best captured variation in envenoming and mortality. We compared the predictive performance of each buffer radius and bilinear interpolation using the log-likelihood ratio test against a null model. These exploratory analyses did not reveal consistent or interpretable improvement with any specific radius. We therefore retained the bilinear interpolation at the centroid as the primary extraction method.

In the exploratory analysis phase, we ignored spatial correlation and used Poisson log-linear models [1] for each of the two outcomes of interest: envenoming bite incidence and mortality. As our objective was to predict the burden rather than to estimate covariate effects (i.e. this is a prediction problem rather than an estimation problem), the explanatory variables with the greatest ability to explain variability in the outcomes were prioritised for model building. We initially implemented a forward variable selection procedure, adding covariates sequentially and retaining them only when they yielded a statistically significant improvement in model fit, as assessed by log-likelihood ratio tests at the conventional 5% significance level. The selected variables were then evaluated within the geostatistical modelling framework. This strategy allowed us to identify covariates with the strongest predictive value while limiting unnecessary model complexity.

To structure our modelling approach, we grouped the explanatory variables into three categories: climatic, socio-economic and other environmental variables. We conducted explanatory analysis within each group to identify variables associated with envenoming incidence. These selected variables were then incorporated into a single non-spatial multivariable model. We also investigated interactions between variables. To further explore spatial heterogeneity, we introduced landmass (i.e., a factor variable denoting eastern and western landmasses) into the global model and evaluated the importance of landmass and of potential interactions with the explanatory variables.

We used empirical log incidence plots to visually examine the functional form of associations between explanatory variables and outcomes. We then used the corresponding generalised additive models [2] to check for any non-linear associations of explanatory variables with outcomes. When generalised additive models suggested possible non-linearity, we evaluated this further by fitting piece-wise models, with knot positions identified using a log-likelihood-based approach (i.e. selecting the knot at the point where the model achieved the maximum log-likelihood). Although some variables showed visual indications of non-linearity, the splines were not statistically significant when incorporated into the models, nor did they improve model fit. Consequently, no splines were retained, and we assumed linear effects for all variables.

Parameter estimates of the non-spatial model for snakebite envenoming incidence are shown in Table A. Although the Gender Inequality Index was not significant in the non-spatial model, it became significant after accounting for spatial dependence in the geostatistical model and was therefore retained.

Table A: Non-spatial model for snakebite envenoming incidence

| Variable | Estimate | Standard error | Z value | P value* |
| --- | --- | --- | --- | --- |
| Intercept | -5.259e+00 | 1.614e-02 | -325.930 | <2e-16 |
| Source type (community survey - reference) |  |  |  |  |
| Country | -8.291e-01 | 1.408e-02 | -58.902 | <2e-16 |
| Regional | -1.811e+00 | 1.407e-02 | -128.754 | <2e-16 |
| Hospital | -1.186e+00 | 3.159e-02 | -37.546 | <2e-16 |
| Minimum temperature (SD) | -1.082e-01 | 3.836e-04 | -282.036 | <2e-16 |
| Urban population (%) | 3.563e-02 | 8.681e-05 | -410.473 | <2e-16 |
| Gender Inequality Index | -1.104e-02 | 1.097e-02 | -1.006 | 0.314 |

*Parameter estimates, standard errors, and corresponding p-values were derived from a Poisson log-linear model, with statistical significance evaluated via two-sided Wald z-tests (ratio of the estimate to its standard error).

To assess potential multicollinearity, Generalised Variance Inflation Factors (GVIFs) were assessed for all explanatory variables in the non-spatial envenoming model during covariate selection. To ensure comparability between continuous variables and categorical factors with multiple levels, we utilised the adjusted GVIFs (i.e. GVIF^(2*Df)). All adjusted values were low and did not indicate problematic multicollinearity (Table B).

Table B: Assessing multicollinearity among explanatory variables included in the non-spatial model for snakebite envenoming incidence

| Variable | GVIF | Df | GVIF^(2*Df) |
| --- | --- | --- | --- |
| Source type | 1.384378 | 3 | 1.055705 |
| Minimum temperature (SD) | 1.607889 | 1 | 1.268026 |
| Urban population (%) | 2.051838 | 1 | 1.432424 |
| Gender Inequality Index | 2.076567 | 1 | 1.441030 |

Although snakebite deaths constitute a subset of envenomings, not all envenoming data sources provided mortality data, and vice versa. Furthermore, the ratio of envenoming to deaths is likely to vary across regions due to differences in snake species composition, access to healthcare, and treatment availability. Therefore, we developed a separate model for snakebite mortality incidence. Since snakebite deaths are mechanistically related to envenoming incidence, we initially explored estimated envenoming incidence as a candidate explanatory variable during the explanatory analysis phase of mortality model development to examine broader associations. After evaluating its behaviour and recognising the conceptual risks, particularly the endogeneity problem and the propagation of uncertainty from one model into another, we did not retain estimated envenoming incidence in the mortality model. In all other respects, we used the same methodology for both envenoming incidence and mortality. Parameter estimates of the non-spatial model for snakebite mortality incidence are shown in Table C.

Table C: Non-spatial model for snakebite mortality incidence

| Variable | Estimate | Standard error | Z value | P value* |
| --- | --- | --- | --- | --- |
| Intercept | -18.99172 | 0.13521 | -140.459 | < 2e-16 |
| Source type (community survey - reference) |  |  |  |  |
| Country | -1.94830 | 0.07912 | -24.625 | < 2e-16 |
| Regional | -2.95329 | 0.07964 | -37.083 | < 2e-16 |
| Hospital | -0.69801 | 0.14477 | -4.822 | 1.42e-06 |
| Category 1 snake species (maximum richness) | 0.39175 | 0.01322 | 29.631 | < 2e-16 |
| Carbon dioxide emissions per capita (production) | -0.18900 | 0.01043 | -18.122 | < 2e-16 |
| Gender Inequality Index | 13.12764 | 0.15700 | 83.615 | < 2e-16 |

*Parameter estimates, standard errors, and corresponding p-values were derived from a Poisson log-linear model, with statistical significance evaluated via two-sided Wald z-tests (ratio of the estimate to its standard error).

Generalised Variance Inflation Factors were assessed for explanatory variables of the non-spatial mortality model during covariate selection. All adjusted GVIF values of the retained covariates were low and did not indicate problematic multicollinearity (Table D).

Table D: Assessing multicollinearity among explanatory variables included in the non-spatial model for snakebite mortality incidence

| Variable | GVIF | Df | GVIF^(2*Df) |
| --- | --- | --- | --- |
| Source type | 1.726851 | 3 | 1.095324 |
| Category 1 snake species (maximum richness) | 1.638752 | 1 | 1.280137 |
| Carbon dioxide emissions per capita (production) | 1.750521 | 1 | 1.323073 |
| Gender Inequality Index | 1.833766 | 1 | 1.354166 |

# Assessing excess variability

We evaluated the presence of excess zeros by comparing the observed number of zero counts with the number expected under a fitted non‑spatial Poisson log‑linear model. After simulating 500 datasets from this model, the expected numbers of zeros were 6 for envenomings and 61 for deaths, whereas the observed data contained only 3 and 42 zeros, respectively. Because the observed zeros did not exceed the expected number, there was no evidence of zero inflation in our dataset.

Both non-spatial Poisson log-linear models showed large overdispersion (envenomings: residual deviance = 451,583.73, df = 228, P < 0.0001; mortality: residual deviance = 7110, df = 161, P < 0.001). We therefore fitted mixed-effects Poisson log-linear models incorporating country-level random effects. The country-level random-effect variance was strongly supported in both models (Δ deviance 111635.5 for envenoming and 4682.63 for mortality). Accounting for the non-standard null distribution for variance components [3], the evidence for between-country heterogeneity remained highly significant (P<0.001 in both models)

Excess variability was then assessed using the empirical variogram of standardised Pearson residuals from non‑spatial Poisson models.

# Assessing spatial correlation

To assess whether residual spatial correlation remained after accounting for the fixed effects, we examined the empirical variogram of the Pearson residuals from each non-spatial model. This diagnostic evaluates whether observations located close to one another have more similar residuals than would be expected under a model with no spatial structure. Detecting spatial correlation in these residuals indicates that spatial dependence persists and justifies the inclusion of a spatial Gaussian process in the model. This approach follows standard practice in model-based geostatistics (Fig A).

|    A) Variogram analysis of envenoming bite incidence |    B) Variogram analysis of snakebite mortality incidence |
| --- | --- |

Fig A: Variogram of the point predictions of standardised Pearson residuals (black line) with the fitted Matern variogram overlaid (red line) for A) envenoming bites and B) snakebite mortality. Variograms of the point predictions provide evidence against the assumption of spatially uncorrelated standardised Pearson residuals for both envenoming and snakebite mortality.

In both cases, the empirical variogram suggested an underlying pattern of increasing values with increasing distance, indicating residual spatial structure. We therefore used a geostatistical approach for our final models. This approach captures spatially structured and unstructured residual variation, and extra‑Poisson variability, through the latent Gaussian process and the nugget effect, respectively.

# Geostatistical modelling of envenoming bites and snakebite mortality

We adopted a model-based geostatistical framework to estimate envenoming bite incidence and snakebite mortality at each location. Geostatistical models incorporate Gaussian process random effects, which allow the local incidence/mortality to vary flexibly in a continuous spatial manner. The Gaussian process at each location accounts for both spatially structured and unstructured extra‑Poisson variation, thereby explicitly modelling overdispersion. The nugget effect of the latent Gaussian process captures unexplained measurement errors, non-spatial sources of noise, and micro-scale spatial variation occurring at a finer scale than the spatial resolution of the data [4]. While this component also captures residual variability associated with differences among data sources, it does not fully eliminate the possibility of systematic ascertainment differences between country-level, regional-level, hospital-based, and community-based data sources; consequently, some residual bias related to case detection, reporting practices, and healthcare access may remain.

Parameter estimates, incidence mapping and spatial predictions for snakebite envenomings and for mortality were obtained from Poisson log-linear geostatistical models, as follows. At location *x*_i_, *Y_i_* is the number of events (envenomings or deaths) out of *n_i_* individuals at risk, where *n_i_* corresponds to the number sampled, the underlying population of the corresponding administrative unit, or the denominator implied by the published incidence rate when not explicitly reported. The random variables *Y_i_* are independent Poisson random variables conditional on a Gaussian process *S(x)* and independent normally distributed random variables Z_i_ with mean zero and variance *τ^2^*. The conditional expectation$,\lambda\left( x_{i} \right),$of *Y_i_* is given by

$\log\left\{ \lambda\left( x_{i} \right) \right\}=\log\left( n_{i} \right)+d\left( x_{i} \right)^{T}\beta+S\left( x_{i} \right)+Z_{i}$(1)

where *d*(*x_i_*) is the set of explanatory variables associated with location *x_i_*. The Gaussian process *S*(*x*) has mean zero, variance σ^2^ and correlation structure Corr[*S(x), S(xˊ)*] = exp(*-u/φ)*, where *u* is the distance between *x* and *xˊ* and *φ* determines the scale of the spatial correlation. The terms *Z_i_* and *S*(*x_i_*) in (1) capture non-spatial and spatially correlated components of the residual spatial variation in *Y_i_* after adjusting for the covariate effects.

The Gaussian process was modelled using the isotropic Matérn covariance function as implemented in the PrevMap package. The Matérn class is defined by the variance *σ^2^*, scale parameter *ϕ*, and smoothness parameter *κ*. The exponential model is a special case of the Matérn model obtained when *κ* = 0.5. We assessed alternative values of the smoothness parameter ( *κ* = 0.5, 1.5, 2.5) by fitting separate geostatistical models and comparing objective function values. The value *κ* = 0.5 yielded the lowest objective function and was therefore selected for the final analysis. This data‑driven comparison ensured that the chosen covariance structure was supported empirically.

Initial values for regression coefficients and covariance parameters were obtained from the respective non-spatial generalized linear models and least-squares fits to the empirical variogram. A Markov chain Monte Carlo (MCMC) algorithm was used to simulate the samples required for Monte Carlo maximum likelihood estimation. MCMC estimation was performed iteratively until both parameter estimates and the objective function stabilised. Initial fixed-effect estimates were obtained from a Poisson log-linear mode, with Gaussian process parameters informed by an empirical variogram. The first MCM run consisted of 10,000 iterations, including 2,000 burn-in iterations, with thinning every 8 iterations. Subsequent rounds used 80,000 iterations, with 46,000 burn-in and thinning every 4 iterations. This process was repeated (three rounds for the envenomating model and six rounds for the mortality model) until convergence was achieved. Autocorrelograms and trace plots were assessed to check convergence. (Fig B).

|  |  |
| --- | --- |
| (A) Autocorrelogram of the simulated samples for envenoming bite incidence | (B) Trace plot of the simulated samples for snakebite envenoming incidence |
|  |  |
| (C) Autocorrelogram of the simulated samples for snakebite mortality incidence | (D) Trace plot of the simulated samples for snakebite death incidence |

Fig B: Convergence diagnostics: (A) autocorrelogram and (B) trace plot of simulated samples for envenoming bite incidence; (C) autocorrelagram and (D) trace plot of simulated samples for snakebite mortality incidence.

The effective sample sizes of the envenoming bite incidence model were large (mean 2,165; range 1,651–2,853), indicating low autocorrelation and good mixing of the MCMC chains. The Gelman–Rubin statistics [5] were close to 1 (mean 1.0008; range 0.9999–1.0046), indicating strong convergence. The effective sample sizes of the mortality incidence model were large (mean 1,920; range 1,494–2,393), indicating low autocorrelation and good mixing of the MCMC chains. The Gelman–Rubin statistics were close to 1 (mean 1.0008; range 0.9999–1.0052), providing strong evidence of convergence. Parameter estimates from the fitted geostatistical Poisson model for envenoming bite and mortality incidence are presented in Tables 2 and 4, respectively. The empirical relationships between the log incidence and the explanatory variables are illustrated in Fig C for envenoming bites and Fig D for mortality.

Fig C: Empirical log incidence versus explanatory variables of the model.

Fig D: Empirical log mortality incidence versus explanatory variables.

## Sensitivity analysis

Missing values for the covariates were imputed for six countries in envenomings (Central African Republic, French Guiana, Hong Kong, Martinique, Occupied Palestinian Territory, and Taiwan) and for four countries in mortality (Central African Republic, Hong Kong, Occupied Palestinian Territory, and Taiwan). We conducted a sensitivity analysis after excluding all countries with imputed covariates to assess the robustness of our models. Table E and Table F provide geostatistical models for envenomings and mortality, respectively, after excluding all countries with imputed covariates.

Table E: Geostatistical model for envenoming bites after excluding countries with imputed covariates

| Variable | Estimate | Standard error | Z value | P value* |
| --- | --- | --- | --- | --- |
| Intercept | -7.836 | 0.673 | -11.637 | <0.001 |
| Source type (community survey - reference) |  |  |  |  |
| Country | -2.187 | 0.340 | -6.440 | <0.001 |
| Regional | -2.209 | 0.289 | -7.638 | <0.001 |
| Hospital | -1.512 | 0.432 | -3.504 | <0.001 |
| Minimum temperature (SD) | -0.186 | 0.040 | -4.677 | <0.001 |
| Urban population (%) | -0.016 | 0.006 | -2.763 | 0.006 |
| Gender Inequality Index | 4.787 | 0.930 | 5.148 | <0.001 |
| Covariance parameters Matern function (kappa=0.5) | | | | |
| σ^2^ | 1.086 | 0.2231 |  |  |
| φ | 84.917 | 0.0068 |  |  |
| τ^2^ / σ^2^ | 0.681 | 0.9184 |  |  |

*Parameter estimates, standard errors, and corresponding p-values were derived from a model-based geostatistical model. P values for the fixed effects were determined using two-sided Wald z-tests based on the ratio of the estimate to its standard error (z value).

Table F: Geostatistical Poisson model for deaths after excluding countries with imputed covariates

| Variable | Estimate | Standard error | Z value | P value* |
| --- | --- | --- | --- | --- |
| Intercept | -13.548 | 1.145 | -11.829 | <0.001 |
| Source type (community survey - reference) |  |  |  |  |
| Country | -3.391 | 0.535 | -6.341 | <0.001 |
| Regional | -3.339 | 0.495 | -6.746 | <0.001 |
| Hospital | -0.615 | 0.679 | -0.905 | 0.365 |
| Category 1 snake species (maximum richness) | 0.280 | 0.133 | 2.103 | 0.035 |
| Carbon dioxide emissions per capita (production) | -0.177 | 0.088 | -2.010 | 0.044 |
| Gender Inequality Index | 4.468 | 2.145 | 2.083 | 0.037 |
| Covariance parameters Matern function (kappa=0.5) | | | | |
| σ^2^ | 2.336 | 0.116 |  |  |
| φ | 339.139 | 0.001 |  |  |
| τ^2^ / σ^2^ | 0.937 | 0.751 |  |  |

*Parameter estimates, standard errors, and corresponding p-values were derived from a model-based geostatistical model. P values for the fixed effects were determined using two-sided Wald z-tests based on the ratio of the estimate to its standard error (z value).

The sensitivity analyses excluding imputed covariate values yielded results that were consistent with the primary analyses. All covariates retained the same direction of association and statistical interpretation. Most coefficient estimates changed by less than 10%, with the largest difference observed for the hospital-source coefficient in the envenoming model, which increased in absolute magnitude from -1.215 to -1.512. In the mortality model, all coefficient estimates differed by less than 3% from the primary analysis. Overall, these findings indicate that imputing missing covariate values had minimal influence on the model estimates or the study conclusions.

# Assessing the goodness of the fitted models

Model validation was done using Monte Carlo methods based on the variograms. The model was validated by testing evidence against the residual spatial correlation in the data. First, 1000 empirical variograms were simulated under the fitted models, and then 95% confidence intervals were calculated at each spatial distance. Both the empirical variograms obtained from the envenoming incidence and snakebite mortality incidence fall within the respective envelopes of 95% tolerance limits, giving no evidence against the lack of fit (Fig E).

|    A) Envenoming bites |    B) Snakebite mortality |
| --- | --- |

Fig E: Assessing the goodness of the fit of the geostatistical models for envenoming bite incidence and snakebite mortality incidence. The solid line is the empirical variogram of the data. The envelope indicates the 95% confidence bands under the fitted spatial model. The empirical variograms obtained from the envenoming data and mortality data fall within the respective 95% tolerance envelopes.

Because the 95% tolerance envelopes were relatively wider and primarily informative about spatial structure, we complemented the variogram-based diagnostics with an additional assessment of predictive calibration using posterior predictive probability plots.

For each datum *Y_i_*, with observed value *y_i_*, we calculated the predictive probability *p_i_ = P(Y_i_ ≤y_i_)*, where *Y_i_* denotes the posterior predictive distribution for location i. In a well-calibrated model, the *p_i_* should be uniformly distributed on (0,1). Because these probabilities are spatially dependent, formal hypothesis testing is not available; instead, calibration was evaluated by examining the empirical cumulative distribution function of the *p_i_* values, which should be approximately linear. This approach provides a direct assessment of predictive performance of the fitted model.

The empirical CDFs of the envenoming and mortality models closely followed the expected linear relationship under a Uniform(0,1) distribution, indicating satisfactory predictive calibration. This suggests that the observed data are consistent with the predictive distributions generated by the fitted geostatistical models, except in the lower tail, where inaccurate predictions have only a small effect on the global burden prediction (Fig F).

|    A) Envenoming bites |    B) Snakebite mortality |
| --- | --- |

Fig F: Calibration assessment of the geostatistical model. The figures display the Posterior Predictive Calibration of the fitted spatial models for envenoming bites and snakebite mortality. The solid blue line represents the Empirical Cumulative Distribution Function (ECDF) of the predictive probabilities *p_i_*, which denote the probability that a value sampled from the model's predictive distribution is less than or equal to the observed value *y_i_*. The dashed red line represents the identity line, corresponding to a perfect Uniform (0,1) distribution. The close adherence of the empirical curve to the diagonal indicates that the models are well-calibrated, demonstrating that the estimated spatial uncertainty and model parameters characterise the variability in the observed data.

Therefore, considering the variogram-based diagnostics and predictive calibration assessment, we concluded that the fitted geostatistical models are compatible with the observed data.

# Predictive mapping

According to our modelling framework (lines 2-4 in Tables 2 and 4), incidence estimates vary systematically by data-source type. Community-based surveys yielded the highest incidence estimates, followed by hospital-based data and regional-level aggregated data, with the lowest estimates from country-level aggregated data. Country- and regional-level aggregated data produced similar estimates for both envenomings and deaths, while hospital and community-level data yielded comparable estimates for mortality. On this basis, country-level predictions were designated as the lowest estimates, hospital-level predictions as intermediate estimates, and community survey-based predictions as the highest estimates for each country. These systematic differences across data sources reflect heterogeneity in reporting intensity, case ascertainment, and aggregation, with country-level estimates likely arising from low-incidence settings, and hospital- and community-survey-based estimates likely arising from high-incidence settings.

Building on this structure, we generated low- and high-burden estimates for each country under different assumptions about plausible incidence and extent of data under-reporting. For high-income countries (HICs), we assumed that snakebite incidence is generally low and that under-reporting is relatively limited, given more complete reporting systems and higher health-seeking behaviour. Accordingly, country-level estimates were taken to represent the lowest plausible burden for all HICs, and no additional correction for under-reporting was applied; the same estimate is therefore reported for both the low and the high estimates. For all other countries, where the incidence is typically higher, and under-reporting is more likely due to limited surveillance infrastructure and lower health‑seeking behaviour, we defined the low estimate using hospital‑level predictions, which partially correct for under‑reporting while avoiding overestimation, and the high estimate using community-based survey predictions, intended to capture the full extent of under‑reporting in these settings. Please note that these low and high estimates represent alternative scenario assumptions and are not intended to represent statistical uncertainty bounds (e.g. confidence or predictive intervals) for a given region or country.

Our fitted models used country-wide average values of the explanatory variables and generated country-wide predictions as follows. Firstly, plugin predictions were made for each country by drawing 1000 samples from the posterior predictive distribution for each country. Next, the average of these 1,000 samples was used as our point prediction. Finally, the range from the 25^th^ to the 975^th^ of the 1000 ordered values defined the 95% predictive interval. These predictive intervals account for uncertainty arising from the latent spatial Gaussian process and data‑level uncertainty introduced by the stochastic Poisson likelihood, which reflects random variation in observed counts. In addition, the predictive intervals reflect uncertainty associated with the availability and quality of the underlying empirical data. Consequently, estimates for countries and regions with sparse or limited data are generally associated with wider predictive intervals, reflecting both statistical uncertainty within the modelling framework and limitations in the underlying evidence base.

The geographical extent of categories one and two snakes was used to identify the population at risk, *n*, of envenomings and deaths in each country. This was achieved by overlaying the snake richness raster file on top of the population raster files. The same population raster file was used to calculate the total population, *N*, for each country. Point predictions of individual-level risk, *r*, were multiplied by the size of the population at risk, *n*, to obtain the predicted numbers, *rn*, of annual envenomings and deaths for each country. Annual incidence rates per 100,000 at risk were calculated as I = 100,000rn/N. Note that *n* = *N* when the snake distribution is not geographically restricted for a given country (i.e. I* = 100,000r). Note that the predicted numbers and incidence rates were derived at the country level and did not account for within-country spatial variation in snakebite risk or population distribution. Because estimated numbers are obtained by multiplying incidence by population, countries with large populations may have relatively high numbers of cases despite low incidence. This reflects a population‑size effect, and incidence rates should therefore be used to compare risk across countries.

All analyses were performed in the R programming language version 4.5.1 [6]. Geostatistical modelling, including Monte Carlo maximum likelihood estimation were implemented using the PrevMap package version 1.2.2 [7].

# References

1.	McCullagh P, Nelder JA. Generalized Linear Models. Second. London: Chapman and Hall; 1989.

2.	Wood SN. Generalized Additive Models: An Introduction with R. Second. Boca Raton: Chapman and Hall/CRC; 2017.

3.	Self SG, Liang K-Y. Asymptotic Properties of Maximum Likelihood Estimators and Likelihood Ratio Tests under Nonstandard Conditions. J Am Stat Assoc. 1987 [cited 11 Feb 2025]. Available: https://www.tandfonline.com/doi/abs/10.1080/01621459.1987.10478472

4.	Diggle PJ, Tawn JA, Moyeed RA. Model-Based Geostatistics. J R Stat Soc Ser C Appl Stat. 1998;47: 299–350. doi:10.1111/1467-9876.00113

5.	Gelman A, Rubin DB. Inference from Iterative Simulation Using Multiple Sequences. Stat Sci. 1992;7: 457–472. doi:10.1214/ss/1177011136

6.	R Core Team. R: A Language and Environment for Statistical Computing. Vienna, Austria: R Foundation for Statistical Computing; 2025. Available: https://www.R-project.org/

7.	Giorgi E, Diggle P. PrevMap: An R Package for Prevalence Mapping. J Stat Softw. 2017;78. doi:10.18637/jss.v078.i08
