# Supplementary material for: Estimates of global burden of snakebite: A literature review and geostatistical modelling study
Source: PLoS Med. 2026 Aug 25;23(8):e1005201. doi: 10.1371/journal.pmed.1005201 (PMC13505955; doi:10.1371/journal.pmed.1005201)
Supplement: Supplementary file 3 [file pmed.1005201.s004.docx]

Table A: Estimated envenoming incidence per 100,000 people and number of annual numbers of envenomings (95% Predictive Interval) derived from country‑level estimates (reflecting no correction for under‑reporting), derived from hospital‑based estimates (reflecting partial correction for under‑reporting), and derived from community‑based survey estimates (reflecting extensive correction for under‑reporting). Please note that no corrections have been made for high-income countries (HICs) in hospital-based and survey-based estimates. *Estimates presented in the manuscript as low estimate and high estimate scenarios.

|  | **Country level estimates**  **(no correction for under‑reporting)** | | **Hospital level estimates**  **(partial correction for under‑reporting) *** | | **Survey level estimates**  **(extensive correction for under‑reporting) *** | |
| --- | --- | --- | --- | --- | --- | --- |
| **Country** | **Incidence/100,000** | **Annual envenomings** | **Incidence/100,000** | **Annual envenomings** | **Incidence/100,000** | **Annual envenomings** |
| Papua New Guinea | 86.9 (71.3 - 106.1) | 7,750 (6,400 - 9,500) | 231.0 (190.2 - 279.7) | 20,700 (17,000 - 25,000) | 779.3 (640.6 - 946.7) | 69,700 (57,300 - 84,700) |
| Liberia | 52.3 (37.9 - 70.4) | 2,150 (1,550 - 2,900) | 138.9 (101.2 - 186.7) | 5,750 (4,200 - 7,700) | 468.5 (340.8 - 636.4) | 19,300 (14,100 - 26,300) |
| South Sudan | 52.3 (46.3 - 59.3) | 8,750 (7,750 - 9,900) | 138.8 (124.6 - 156.7) | 23,200 (20,800 - 26,200) | 468.3 (417.8 - 527.6) | 78,200 (69,800 - 88,100) |
| Sierra Leone | 48.9 (37.1 - 63.6) | 2,950 (2,250 - 3,850) | 129.7 (100.4 - 168.6) | 7,900 (6,100 - 10,300) | 438.9 (334.4 - 563.2) | 26,700 (20,300 - 34,200) |
| Malawi | 46.9 (34.1 - 63.5) | 8,750 (6,350 - 11,800) | 124.1 (93.1 - 167.6) | 23,200 (17,400 - 31,300) | 419.2 (312.1 - 553.4) | 78,200 (58,200 - 103,000) |
| Uganda | 46.8 (39.3 - 55.2) | 19,900 (16,700 - 23,500) | 125.2 (105.4 - 147.6) | 53,200 (44,800 - 62,800) | 421.6 (353.5 - 501.0) | 179,000 (150,000 - 213,000) |
| Democratic Republic of the Congo | 45.8 (42.9 - 48.6) | 52,100 (48,800 - 55,200) | 121.8 (114.2 - 129.4) | 139,000 (130,000 - 147,000) | 410.5 (387.2 - 435.0) | 467,000 (440,000 - 495,000) |
| Kenya | 45.7 (40.7 - 51.6) | 25,100 (22,400 - 28,300) | 120.6 (108.4 - 136.1) | 66,300 (59,500 - 74,800) | 407.3 (362.6 - 459.5) | 224,000 (199,000 - 252,000) |
| Guinea | 45.4 (37.2 - 54.9) | 5,600 (4,600 - 6,750) | 121.2 (100.1 - 146.5) | 14,900 (12,300 - 18,000) | 409.4 (337.7 - 494.1) | 50,400 (41,600 - 60,800) |
| Burkina Faso | 42.9 (36.0 - 50.9) | 9,800 (8,200 - 11,600) | 114.7 (96.3 - 138.4) | 26,100 (21,900 - 31,500) | 384.5 (321.0 - 463.2) | 87,600 (73,100 - 106,000) |
| Burundi | 42.3 (32.0 - 55.1) | 4,800 (3,650 - 6,300) | 112.3 (83.6 - 148.5) | 12,800 (9,500 - 16,900) | 376.3 (282.0 - 493.7) | 42,900 (32,100 - 56,300) |
| SÃ£o TomÃ© and Principe | 42.0 (16.5 - 89.6) | 60 (25 - 150) | 108.2 (44.0 - 227.3) | 150 (70 - 350) | 365.9 (149.6 - 773.8) | 550 (250 - 1,200) |
| Guinea-Bissau | 41.6 (30.9 - 55.2) | 750 (550 - 1,000) | 109.6 (79.8 - 148.3) | 1,950 (1,400 - 2,600) | 369.3 (273.4 - 496.3) | 6,550 (4,850 - 8,800) |
| Benin | 40.3 (31.0 - 52.6) | 5,300 (4,050 - 6,900) | 107.3 (81.5 - 137.3) | 14,100 (10,700 - 18,000) | 360.2 (275.8 - 461.3) | 47,200 (36,100 - 60,500) |
| Ivory Coast | 39.4 (33.7 - 45.7) | 10,200 (8,700 - 11,800) | 103.9 (89.8 - 122.3) | 26,900 (23,200 - 31,600) | 350.3 (302.9 - 406.0) | 90,600 (78,400 - 105,000) |
| Togo | 38.7 (29.4 - 50.5) | 3,300 (2,500 - 4,350) | 103.0 (77.7 - 137.8) | 8,850 (6,650 - 11,800) | 344.3 (259.8 - 454.2) | 29,500 (22,300 - 38,900) |
| Central African Republic | 37.6 (32.9 - 42.7) | 2,050 (1,800 - 2,350) | 99.9 (88.5 - 113.4) | 5,450 (4,850 - 6,200) | 337.1 (295.6 - 382.5) | 18,400 (16,100 - 20,900) |
| Nigeria | 34.6 (31.8 - 37.9) | 74,500 (68,500 - 81,500) | 92.0 (84.3 - 100.9) | 198,000 (181,000 - 217,000) | 309.8 (283.5 - 339.0) | 666,000 (610,000 - 729,000) |
| Ethiopia | 34.0 (31.3 - 37.2) | 35,300 (32,500 - 38,600) | 90.7 (83.3 - 98.9) | 94,100 (86,400 - 103,000) | 305.7 (281.9 - 332.7) | 317,000 (292,000 - 345,000) |
| Cambodia | 33.9 (27.8 - 41.3) | 6,600 (5,400 - 8,050) | 89.9 (73.3 - 111.1) | 17,500 (14,300 - 21,600) | 303.5 (247.1 - 370.5) | 59,000 (48,000 - 72,000) |
| Chad | 32.3 (30.1 - 34.8) | 5,350 (5,000 - 5,800) | 85.5 (79.6 - 92.3) | 14,200 (13,200 - 15,300) | 289.1 (267.6 - 311.6) | 48,000 (44,400 - 51,700) |
| Yemen | 31.5 (27.3 - 36.6) | 9,400 (8,150 - 10,900) | 83.5 (71.5 - 96.3) | 24,900 (21,300 - 28,700) | 282.5 (245.0 - 329.2) | 84,200 (73,100 - 98,200) |
| Guyana | 30.5 (24.9 - 36.7) | 200 (150 - 250) | 30.5 (25.1 - 37.0) | 200 (150 - 250) | 30.5 (25.1 - 37.0) | 200 (150 - 250) |
| Niger | 30.0 (27.5 - 32.8) | 7,150 (6,550 - 7,850) | 80.0 (73.2 - 87.0) | 19,100 (17,500 - 20,800) | 269.1 (247.0 - 292.7) | 64,200 (59,000 - 69,900) |
| United Republic of Tanzania | 29.4 (26.9 - 32.0) | 16,400 (15,000 - 17,900) | 77.7 (71.3 - 84.6) | 43,400 (39,900 - 47,200) | 262.0 (240.4 - 284.2) | 146,000 (134,000 - 159,000) |
| Republic of the Congo | 29.0 (24.6 - 34.5) | 1,100 (950 - 1,300) | 77.3 (64.8 - 92.5) | 2,950 (2,500 - 3,550) | 260.5 (220.0 - 309.7) | 9,950 (8,400 - 11,800) |
| Somalia | 28.7 (23.8 - 34.9) | 2,300 (1,950 - 2,800) | 76.5 (62.8 - 91.2) | 6,200 (5,050 - 7,350) | 257.9 (214.1 - 310.5) | 20,800 (17,300 - 25,100) |
| Cameroon | 27.7 (23.9 - 32.2) | 7,800 (6,750 - 9,050) | 73.6 (63.6 - 85.1) | 20,700 (17,900 - 24,000) | 247.6 (213.1 - 288.0) | 69,700 (60,000 - 81,000) |
| Eritrea | 27.3 (18.5 - 39.4) | 1,150 (800 - 1,700) | 71.6 (48.4 - 105.2) | 3,050 (2,050 - 4,500) | 246.2 (167.3 - 369.7) | 10,500 (7,150 - 15,800) |
| Rwanda | 26.0 (20.3 - 33.6) | 3,550 (2,750 - 4,550) | 68.8 (53.5 - 87.6) | 9,350 (7,250 - 11,900) | 234.2 (182.0 - 307.0) | 31,800 (24,700 - 41,700) |
| Sri Lanka | 24.3 (18.9 - 31.1) | 4,900 (3,800 - 6,250) | 64.9 (49.8 - 84.2) | 13,100 (10,000 - 17,000) | 218.3 (167.1 - 281.5) | 44,000 (33,600 - 56,700) |
| Ghana | 23.8 (20.1 - 27.9) | 7,750 (6,550 - 9,100) | 63.4 (53.8 - 74.5) | 20,700 (17,500 - 24,300) | 214.0 (180.8 - 253.3) | 69,700 (58,900 - 82,600) |
| Equatorial Guinea | 23.6 (15.3 - 35.2) | 300 (200 - 400) | 62.5 (40.0 - 93.4) | 750 (450 - 1,100) | 213.3 (138.0 - 315.9) | 2,550 (1,650 - 3,750) |
| Gambia | 23.1 (16.5 - 32.3) | 500 (350 - 700) | 62.6 (43.9 - 89.1) | 1,350 (950 - 1,900) | 207.1 (146.9 - 286.7) | 4,400 (3,150 - 6,100) |
| East Timor | 21.8 (13.9 - 32.6) | 300 (200 - 450) | 57.7 (38.1 - 86.8) | 800 (550 - 1,200) | 197.3 (127.9 - 301.8) | 2,750 (1,750 - 4,150) |
| Mozambique | 21.2 (18.6 - 23.8) | 6,400 (5,650 - 7,200) | 56.0 (50.3 - 63.5) | 17,000 (15,200 - 19,200) | 189.3 (167.0 - 213.1) | 57,300 (50,600 - 64,500) |
| Lesotho | 21.1 (16.2 - 27.9) | 400 (300 - 500) | 56.3 (42.0 - 74.6) | 1,050 (800 - 1,400) | 189.5 (144.9 - 247.0) | 3,550 (2,700 - 4,600) |
| Zambia | 20.8 (18.7 - 23.1) | 3,900 (3,500 - 4,300) | 55.3 (49.5 - 61.7) | 10,300 (9,250 - 11,500) | 186.2 (166.3 - 207.8) | 34,800 (31,100 - 38,800) |
| Zimbabwe | 20.7 (18.2 - 23.5) | 2,950 (2,600 - 3,350) | 55.0 (48.8 - 62.9) | 7,850 (7,000 - 9,000) | 185.5 (160.8 - 211.0) | 26,500 (23,000 - 30,200) |
| eSwatini | 20.5 (16.4 - 26.2) | 200 (200 - 300) | 54.7 (43.2 - 68.6) | 600 (450 - 750) | 183.7 (147.3 - 226.6) | 2,000 (1,600 - 2,450) |
| Indonesia | 20.2 (18.0 - 22.5) | 54,700 (48,700 - 61,000) | 53.7 (48.0 - 59.9) | 145,000 (130,000 - 162,000) | 180.9 (161.5 - 202.1) | 490,000 (438,000 - 548,000) |
| Mali | 19.5 (17.8 - 21.5) | 4,550 (4,150 - 5,050) | 52.0 (47.3 - 57.6) | 12,100 (11,000 - 13,400) | 175.7 (159.7 - 194.2) | 41,000 (37,300 - 45,300) |
| Senegal | 19.3 (15.9 - 23.6) | 3,100 (2,550 - 3,800) | 51.4 (42.5 - 62.4) | 8,300 (6,850 - 10,100) | 173.1 (143.4 - 208.6) | 27,900 (23,100 - 33,600) |
| Somaliland | 19.3 (15.8 - 23.7) | 550 (450 - 650) | 50.7 (40.9 - 62.3) | 1,400 (1,150 - 1,700) | 171.9 (139.9 - 209.5) | 4,750 (3,850 - 5,800) |
| Belize | 19.2 (14.4 - 25.1) | 80 (60 - 100) | 50.7 (38.3 - 67.3) | 200 (150 - 250) | 170.3 (131.4 - 223.9) | 700 (550 - 900) |
| Sudan | 19.1 (18.0 - 20.3) | 8,150 (7,700 - 8,650) | 50.7 (47.4 - 53.9) | 21,600 (20,200 - 23,000) | 170.8 (160.4 - 182.1) | 72,800 (68,300 - 77,600) |
| Laos | 17.8 (13.8 - 23.1) | 1,350 (1,050 - 1,750) | 47.8 (36.6 - 60.7) | 3,600 (2,750 - 4,550) | 161.0 (124.0 - 208.1) | 12,100 (9,350 - 15,700) |
| Venezuela | 17.6 (15.9 - 19.4) | 5,450 (4,900 - 6,000) | 46.8 (42.2 - 51.9) | 14,500 (13,100 - 16,100) | 157.7 (141.8 - 175.0) | 48,800 (43,900 - 54,200) |
| Mauritania | 16.9 (15.5 - 18.5) | 750 (700 - 800) | 45.0 (41.2 - 49.2) | 1,950 (1,800 - 2,150) | 151.6 (138.6 - 165.1) | 6,600 (6,050 - 7,200) |
| Gabon | 16.6 (13.4 - 20.5) | 450 (350 - 550) | 44.1 (36.0 - 54.5) | 1,200 (1,000 - 1,500) | 148.3 (119.8 - 180.6) | 4,100 (3,300 - 5,000) |
| Myanmar | 16.3 (14.1 - 18.7) | 7,750 (6,700 - 8,850) | 43.3 (37.4 - 49.6) | 20,500 (17,700 - 23,500) | 146.5 (126.1 - 169.9) | 69,400 (59,700 - 80,500) |
| Panama | 16.3 (12.0 - 22.0) | 650 (500 - 900) | 16.6 (12.1 - 22.7) | 650 (500 - 900) | 16.6 (12.1 - 22.7) | 650 (500 - 900) |
| Philippines | 16.3 (11.7 - 22.1) | 16,000 (11,500 - 21,700) | 43.3 (31.5 - 60.1) | 42,600 (31,000 - 59,100) | 145.8 (104.6 - 204.8) | 143,000 (103,000 - 201,000) |
| Suriname | 16.3 (13.4 - 20.0) | 100 (80 - 100) | 43.4 (35.2 - 52.9) | 250 (200 - 350) | 146.0 (119.4 - 180.9) | 900 (750 - 1,100) |
| Saint Lucia | 16.2 (12.4 - 20.7) | 20 (15 - 25) | 43.0 (33.3 - 54.0) | 60 (45 - 70) | 145.4 (113.0 - 183.1) | 200 (150 - 250) |
| Afghanistan | 16.1 (14.1 - 18.4) | 4,900 (4,300 - 5,600) | 42.7 (37.8 - 48.1) | 13,000 (11,500 - 14,600) | 144.2 (127.9 - 162.4) | 43,700 (38,800 - 49,300) |
| Angola | 15.7 (14.5 - 17.1) | 5,550 (5,100 - 6,050) | 41.7 (38.6 - 44.8) | 14,700 (13,600 - 15,800) | 140.4 (130.4 - 152.1) | 49,500 (45,900 - 53,600) |
| Guatemala | 15.6 (12.0 - 20.5) | 2,850 (2,200 - 3,750) | 41.3 (31.6 - 53.9) | 7,550 (5,750 - 9,850) | 139.5 (105.6 - 187.5) | 25,500 (19,300 - 34,300) |
| Nepal | 15.2 (11.6 - 20.0) | 6,300 (4,850 - 8,300) | 40.2 (30.4 - 52.9) | 16,700 (12,600 - 22,000) | 136.8 (103.2 - 176.7) | 56,800 (42,900 - 73,400) |
| Bangladesh | 14.8 (11.5 - 19.4) | 23,900 (18,600 - 31,200) | 39.4 (29.9 - 50.0) | 63,500 (48,300 - 80,600) | 133.0 (103.3 - 172.0) | 214,000 (166,000 - 277,000) |
| Nicaragua | 13.9 (10.7 - 17.9) | 950 (750 - 1,200) | 37.0 (28.7 - 46.6) | 2,500 (1,950 - 3,200) | 124.4 (96.9 - 162.6) | 8,500 (6,600 - 11,100) |
| Vietnam | 13.0 (10.6 - 16.3) | 12,500 (10,200 - 15,700) | 34.7 (27.3 - 43.7) | 33,500 (26,300 - 42,100) | 117.1 (91.6 - 149.5) | 113,000 (88,200 - 144,000) |
| Western Sahara | 12.6 (6.5 - 22.6) | 8 (4 - 15) | 33.4 (16.7 - 58.3) | 20 (10 - 35) | 113.7 (58.5 - 214.3) | 70 (35 - 150) |
| Honduras | 11.9 (9.1 - 15.5) | 1,150 (850 - 1,450) | 31.5 (24.3 - 41.6) | 3,000 (2,300 - 3,950) | 107.0 (79.9 - 142.7) | 10,100 (7,550 - 13,500) |
| Colombia | 11.7 (10.6 - 12.9) | 7,350 (6,650 - 8,050) | 31.3 (28.4 - 34.5) | 19,600 (17,800 - 21,600) | 105.4 (95.7 - 115.7) | 66,100 (60,000 - 72,500) |
| India | 11.0 (10.3 - 11.8) | 154,000 (144,000 - 164,000) | 29.3 (27.5 - 31.2) | 409,000 (384,000 - 435,000) | 98.7 (92.3 - 105.3) | 1,378,000 (1,290,000 - 1,471,000) |
| Ecuador | 10.2 (7.5 - 13.7) | 1,750 (1,300 - 2,400) | 26.6 (19.8 - 34.9) | 4,650 (3,450 - 6,050) | 90.3 (66.3 - 122.3) | 15,700 (11,500 - 21,200) |
| Djibouti | 9.7 (7.3 - 12.6) | 100 (70 - 150) | 25.9 (20.0 - 34.1) | 250 (200 - 350) | 88.0 (68.3 - 114.1) | 900 (700 - 1,150) |
| Namibia | 9.6 (8.6 - 10.7) | 250 (200 - 250) | 25.5 (22.9 - 28.5) | 650 (550 - 700) | 85.8 (76.6 - 96.3) | 2,150 (1,900 - 2,400) |
| Trinidad and Tobago | 9.4 (6.1 - 14.6) | 100 (80 - 200) | 9.4 (6.0 - 14.8) | 100 (80 - 200) | 9.4 (6.0 - 14.8) | 100 (80 - 200) |
| Thailand | 8.9 (7.5 - 10.4) | 6,550 (5,550 - 7,650) | 23.6 (20.2 - 27.5) | 17,400 (14,900 - 20,300) | 79.5 (68.2 - 92.7) | 58,600 (50,300 - 68,400) |
| Paraguay | 8.8 (7.5 - 10.2) | 650 (550 - 750) | 23.4 (19.9 - 27.0) | 1,700 (1,450 - 2,000) | 79.0 (67.6 - 91.6) | 5,800 (4,950 - 6,700) |
| Brazil | 8.6 (8.3 - 8.9) | 18,100 (17,400 - 18,700) | 22.9 (22.1 - 23.7) | 48,000 (46,400 - 49,600) | 77.1 (74.4 - 80.0) | 162,000 (156,000 - 168,000) |
| Pakistan | 8.4 (7.4 - 9.4) | 19,200 (17,100 - 21,600) | 22.2 (19.7 - 25.0) | 51,100 (45,400 - 57,500) | 74.5 (66.3 - 83.4) | 171,000 (152,000 - 192,000) |
| Morocco | 7.6 (6.3 - 9.0) | 2,700 (2,250 - 3,250) | 20.0 (16.5 - 24.0) | 7,150 (5,900 - 8,600) | 67.4 (56.1 - 80.5) | 24,200 (20,100 - 28,900) |
| Peru | 7.5 (6.9 - 8.2) | 2,650 (2,400 - 2,900) | 19.9 (18.2 - 21.8) | 7,000 (6,400 - 7,650) | 67.1 (61.5 - 73.1) | 23,600 (21,600 - 25,700) |
| Botswana | 7.3 (6.5 - 8.3) | 150 (150 - 200) | 19.4 (17.2 - 21.6) | 450 (400 - 500) | 65.6 (58.4 - 74.2) | 1,550 (1,400 - 1,750) |
| Brunei | 7.0 (4.9 - 10.1) | 30 (20 - 40) | 7.1 (5.1 - 10.2) | 30 (20 - 40) | 7.1 (5.1 - 10.2) | 30 (20 - 40) |
| Iraq | 6.4 (5.6 - 7.3) | 2,850 (2,450 - 3,250) | 16.9 (14.7 - 19.6) | 7,500 (6,550 - 8,700) | 57.0 (49.3 - 65.2) | 25,300 (21,900 - 29,000) |
| Egypt | 6.2 (5.7 - 6.7) | 5,800 (5,400 - 6,300) | 16.4 (15.2 - 17.7) | 15,500 (14,400 - 16,700) | 55.2 (51.1 - 59.5) | 52,100 (48,300 - 56,200) |
| Costa Rica | 6.1 (4.5 - 8.2) | 300 (200 - 400) | 6.1 (4.3 - 8.1) | 300 (200 - 400) | 6.1 (4.3 - 8.1) | 300 (200 - 400) |
| Malaysia | 5.6 (4.3 - 7.4) | 1,850 (1,400 - 2,450) | 15.0 (11.3 - 20.1) | 4,950 (3,700 - 6,600) | 50.2 (37.9 - 65.1) | 16,500 (12,400 - 21,400) |
| Bhutan | 5.4 (4.2 - 6.9) | 50 (40 - 70) | 14.6 (11.4 - 18.8) | 150 (100 - 200) | 49.2 (37.9 - 63.5) | 450 (350 - 600) |
| South Africa | 5.4 (4.8 - 6.3) | 3,250 (2,850 - 3,750) | 14.4 (12.5 - 16.5) | 8,600 (7,450 - 9,850) | 48.6 (42.5 - 56.1) | 28,900 (25,300 - 33,500) |
| Bolivia | 5.1 (4.6 - 5.6) | 600 (550 - 650) | 13.6 (12.3 - 15.0) | 1,600 (1,450 - 1,800) | 45.8 (41.6 - 50.5) | 5,450 (4,900 - 5,950) |
| Syria | 4.8 (3.9 - 5.9) | 1,250 (1,050 - 1,550) | 12.8 (10.4 - 15.8) | 3,400 (2,750 - 4,200) | 43.3 (35.4 - 52.9) | 11,500 (9,400 - 14,000) |
| El Salvador | 4.6 (3.6 - 5.9) | 300 (250 - 400) | 12.3 (9.6 - 15.9) | 800 (650 - 1,050) | 41.9 (32.3 - 53.5) | 2,800 (2,150 - 3,550) |
| Mexico | 4.4 (4.0 - 4.8) | 6,100 (5,600 - 6,700) | 11.7 (10.7 - 12.8) | 16,300 (14,900 - 17,900) | 39.4 (36.1 - 43.2) | 55,000 (50,500 - 60,300) |
| Iran | 4.2 (3.9 - 4.6) | 3,400 (3,150 - 3,650) | 11.3 (10.5 - 12.1) | 9,000 (8,400 - 9,700) | 38.0 (35.3 - 40.8) | 30,300 (28,200 - 32,600) |
| Palestine | 3.7 (2.6 - 5.0) | 250 (150 - 300) | 9.6 (7.0 - 13.2) | 600 (450 - 850) | 32.7 (24.1 - 44.8) | 2,050 (1,550 - 2,850) |
| Lebanon | 3.5 (2.5 - 4.9) | 300 (200 - 400) | 9.3 (6.7 - 12.9) | 800 (550 - 1,100) | 31.1 (22.3 - 43.8) | 2,600 (1,900 - 3,700) |
| French Guiana | 3.3 (2.6 - 4.3) | 10 (8 - 15) | 3.3 (2.5 - 4.2) | 10 (8 - 15) | 3.3 (2.5 - 4.2) | 10 (8 - 15) |
| Jordan | 3.3 (2.5 - 4.4) | 250 (150 - 300) | 8.7 (6.5 - 11.8) | 600 (450 - 850) | 29.3 (21.9 - 39.4) | 2,100 (1,550 - 2,800) |
| Kyrgyzstan | 3.1 (2.4 - 3.8) | 200 (150 - 200) | 8.1 (6.6 - 10.1) | 450 (400 - 600) | 27.3 (22.0 - 33.7) | 1,600 (1,300 - 1,950) |
| Cyprus | 2.9 (2.2 - 3.9) | 25 (20 - 35) | 2.9 (2.2 - 3.8) | 25 (20 - 35) | 2.9 (2.2 - 3.8) | 25 (20 - 35) |
| Northern Cyprus | 2.9 (2.0 - 4.1) | 10 (7 - 15) | 2.9 (2.0 - 4.2) | 10 (7 - 15) | 2.9 (2.0 - 4.2) | 10 (7 - 15) |
| Algeria | 2.8 (2.6 - 2.9) | 1,200 (1,100 - 1,250) | 7.4 (6.9 - 7.8) | 3,200 (3,000 - 3,350) | 24.9 (23.5 - 26.4) | 10,700 (10,100 - 11,400) |
| Tajikistan | 2.7 (2.1 - 3.5) | 250 (200 - 350) | 7.2 (5.6 - 9.7) | 700 (500 - 900) | 24.5 (18.8 - 32.2) | 2,300 (1,750 - 3,000) |
| Turkmenistan | 2.7 (2.3 - 3.1) | 250 (250 - 300) | 7.1 (6.1 - 8.2) | 700 (600 - 800) | 23.8 (20.6 - 27.3) | 2,350 (2,050 - 2,700) |
| Martinique (France) | 2.4 (1.8 - 3.1) | 8 (6 - 10) | 2.4 (1.9 - 3.1) | 8 (7 - 10) | 2.4 (1.9 - 3.1) | 8 (7 - 10) |
| Azerbaijan | 2.3 (1.7 - 3.2) | 250 (150 - 300) | 6.1 (4.4 - 8.1) | 600 (450 - 850) | 20.8 (15.0 - 28.8) | 2,100 (1,550 - 2,950) |
| Oman | 2.3 (1.8 - 2.8) | 80 (70 - 100) | 2.3 (1.9 - 2.7) | 80 (70 - 100) | 2.3 (1.9 - 2.7) | 80 (70 - 100) |
| Georgia | 2.1 (1.5 - 2.8) | 80 (50 - 100) | 5.5 (4.0 - 7.6) | 200 (150 - 300) | 18.8 (13.6 - 25.9) | 700 (500 - 950) |
| Liechtenstein | 2.1 (1.6 - 2.7) | 1 (0 - 1) | 2.0 (1.5 - 2.7) | 1 (0 - 1) | 2.0 (1.5 - 2.7) | 1 (0 - 1) |
| Argentina | 2.0 (1.8 - 2.1) | 900 (850 - 950) | 5.2 (4.9 - 5.5) | 2,350 (2,200 - 2,500) | 17.6 (16.5 - 18.8) | 7,900 (7,400 - 8,450) |
| Uruguay | 2.0 (1.6 - 2.4) | 60 (50 - 80) | 2.0 (1.6 - 2.4) | 60 (50 - 80) | 2.0 (1.6 - 2.4) | 60 (50 - 80) |
| Macao S.A.R | 1.9 (1.5 - 2.5) | 3 (2 - 4) | 1.9 (1.4 - 2.5) | 3 (2 - 4) | 1.9 (1.4 - 2.5) | 3 (2 - 4) |
| Tunisia | 1.9 (1.5 - 2.3) | 200 (150 - 250) | 5.0 (3.9 - 6.3) | 550 (450 - 700) | 16.9 (13.5 - 21.3) | 1,850 (1,450 - 2,300) |
| Bosnia and Herzegovina | 1.8 (1.4 - 2.3) | 60 (45 - 80) | 4.8 (3.6 - 6.5) | 150 (100 - 200) | 16.2 (12.1 - 21.8) | 550 (400 - 750) |
| Libya | 1.8 (1.7 - 1.9) | 100 (100 - 150) | 4.8 (4.5 - 5.2) | 350 (300 - 350) | 16.3 (15.2 - 17.5) | 1,100 (1,050 - 1,200) |
| Portugal | 1.7 (0.7 - 3.7) | 150 (60 - 350) | 1.7 (0.6 - 3.5) | 150 (60 - 350) | 1.7 (0.6 - 3.5) | 150 (60 - 350) |
| Turkey | 1.7 (1.6 - 1.9) | 1,350 (1,250 - 1,450) | 4.6 (4.2 - 5.1) | 3,550 (3,250 - 3,900) | 15.6 (14.1 - 17.3) | 12,000 (10,900 - 13,300) |
| Taiwan | 1.6 (1.1 - 2.4) | 350 (250 - 550) | 1.6 (1.1 - 2.4) | 350 (250 - 550) | 1.6 (1.1 - 2.4) | 350 (250 - 550) |
| Albania | 1.5 (1.2 - 2.0) | 40 (30 - 60) | 4.1 (3.1 - 5.3) | 100 (80 - 150) | 13.7 (10.3 - 18.2) | 350 (300 - 500) |
| Armenia | 1.5 (1.1 - 2.1) | 40 (30 - 60) | 4.0 (2.9 - 5.6) | 100 (80 - 150) | 13.6 (9.6 - 19.4) | 400 (250 - 550) |
| North Macedonia | 1.5 (1.2 - 1.9) | 30 (25 - 40) | 3.9 (3.1 - 5.0) | 80 (70 - 100) | 13.2 (10.3 - 16.7) | 300 (200 - 350) |
| Saudi Arabia | 1.5 (1.4 - 1.7) | 550 (500 - 600) | 1.5 (1.4 - 1.6) | 550 (550 - 600) | 1.5 (1.4 - 1.6) | 550 (550 - 600) |
| Montenegro | 1.4 (1.0 - 1.9) | 8 (6 - 10) | 3.7 (2.7 - 5.1) | 20 (15 - 30) | 12.6 (9.1 - 17.1) | 80 (50 - 100) |
| United Kingdom | 1.4 (1.1 - 1.9) | 950 (750 - 1,200) | 1.4 (1.1 - 1.9) | 950 (750 - 1,200) | 1.4 (1.1 - 1.9) | 950 (750 - 1,200) |
| Kosovo | 1.3 (0.9 - 1.8) | 25 (20 - 35) | 3.5 (2.6 - 4.7) | 70 (50 - 100) | 11.6 (8.6 - 15.8) | 250 (200 - 350) |
| Poland | 1.3 (1.1 - 1.5) | 500 (450 - 550) | 1.3 (1.1 - 1.4) | 500 (450 - 550) | 1.3 (1.1 - 1.4) | 500 (450 - 550) |
| Slovakia | 1.3 (1.0 - 1.6) | 70 (50 - 90) | 1.3 (1.0 - 1.7) | 70 (50 - 90) | 1.3 (1.0 - 1.7) | 70 (50 - 90) |
| Uzbekistan | 1.3 (1.1 - 1.5) | 450 (350 - 500) | 3.4 (2.8 - 4.0) | 1,150 (950 - 1,350) | 11.4 (9.5 - 13.4) | 3,850 (3,200 - 4,500) |
| Bahrain | 1.2 (0.9 - 1.6) | 20 (15 - 25) | 1.2 (0.9 - 1.6) | 20 (15 - 25) | 1.2 (0.9 - 1.6) | 20 (15 - 25) |
| Bulgaria | 1.2 (0.9 - 1.5) | 90 (70 - 100) | 1.2 (0.9 - 1.5) | 90 (70 - 100) | 1.2 (0.9 - 1.5) | 90 (70 - 100) |
| Croatia | 1.2 (0.8 - 1.9) | 50 (30 - 70) | 1.2 (0.8 - 1.9) | 45 (30 - 70) | 1.2 (0.8 - 1.9) | 45 (30 - 70) |
| Czechia | 1.2 (0.9 - 1.5) | 100 (90 - 150) | 1.2 (0.9 - 1.5) | 150 (100 - 150) | 1.2 (0.9 - 1.5) | 150 (100 - 150) |
| Latvia | 1.2 (0.9 - 1.6) | 30 (20 - 40) | 1.2 (0.9 - 1.6) | 30 (20 - 40) | 1.2 (0.9 - 1.6) | 30 (20 - 40) |
| Slovenia | 1.2 (0.9 - 1.6) | 25 (20 - 35) | 1.2 (0.9 - 1.6) | 25 (20 - 35) | 1.2 (0.9 - 1.6) | 25 (20 - 35) |
| Ukraine | 1.2 (1.0 - 1.3) | 500 (450 - 550) | 3.1 (2.8 - 3.6) | 1,300 (1,150 - 1,450) | 10.6 (9.4 - 12.0) | 4,350 (3,850 - 4,950) |
| France | 1.1 (1.0 - 1.3) | 750 (650 - 850) | 1.1 (1.0 - 1.3) | 750 (650 - 850) | 1.1 (1.0 - 1.3) | 750 (650 - 850) |
| Lithuania | 1.1 (0.9 - 1.5) | 30 (25 - 40) | 1.1 (0.9 - 1.5) | 30 (25 - 40) | 1.1 (0.9 - 1.5) | 30 (25 - 40) |
| Austria | 1.0 (0.7 - 1.3) | 90 (60 - 100) | 1.0 (0.7 - 1.3) | 90 (70 - 100) | 1.0 (0.7 - 1.3) | 90 (70 - 100) |
| Greece | 1.0 (0.6 - 1.5) | 100 (60 - 150) | 1.0 (0.6 - 1.5) | 100 (60 - 150) | 1.0 (0.6 - 1.5) | 100 (60 - 150) |
| Qatar | 1.0 (0.8 - 1.2) | 60 (50 - 80) | 1.0 (0.7 - 1.3) | 60 (45 - 80) | 1.0 (0.7 - 1.3) | 60 (45 - 80) |
| Andorra | 0.9 (0.7 - 1.1) | 1 (1 - 1) | 0.9 (0.7 - 1.1) | 1 (1 - 1) | 0.9 (0.7 - 1.1) | 1 (1 - 1) |
| Australia | 0.9 (0.8 - 0.9) | 200 (150 - 200) | 0.9 (0.8 - 0.9) | 200 (150 - 200) | 0.9 (0.8 - 0.9) | 200 (150 - 200) |
| Germany | 0.9 (0.8 - 1.1) | 700 (650 - 850) | 0.9 (0.8 - 1.1) | 700 (650 - 850) | 0.9 (0.8 - 1.1) | 700 (650 - 850) |
| Spain | 0.9 (0.7 - 1.1) | 450 (350 - 550) | 0.9 (0.7 - 1.1) | 450 (350 - 550) | 0.9 (0.7 - 1.1) | 450 (350 - 550) |
| United States of America | 0.9 (0.9 - 0.9) | 3,000 (2,900 - 3,100) | 0.9 (0.9 - 0.9) | 3,000 (2,950 - 3,100) | 0.9 (0.9 - 0.9) | 3,000 (2,950 - 3,100) |
| Belarus | 0.8 (0.6 - 1.0) | 70 (60 - 90) | 2.1 (1.7 - 2.6) | 200 (150 - 250) | 7.0 (5.7 - 8.7) | 650 (500 - 800) |
| Belgium | 0.8 (0.6 - 1.1) | 100 (70 - 150) | 0.8 (0.6 - 1.1) | 100 (80 - 150) | 0.8 (0.6 - 1.1) | 100 (80 - 150) |
| China | 0.8 (0.7 - 0.8) | 10,900 (10,500 - 11,300) | 2.0 (2.0 - 2.1) | 28,900 (27,900 - 29,900) | 6.9 (6.6 - 7.1) | 97,400 (94,100 - 100,000) |
| Denmark | 0.8 (0.5 - 1.1) | 40 (30 - 60) | 0.8 (0.5 - 1.1) | 40 (25 - 60) | 0.8 (0.5 - 1.1) | 40 (25 - 60) |
| Estonia | 0.8 (0.6 - 1.1) | 10 (8 - 15) | 0.8 (0.6 - 1.1) | 10 (8 - 15) | 0.8 (0.6 - 1.1) | 10 (8 - 15) |
| Hong Kong S.A.R. | 0.8 (0.6 - 1.0) | 50 (40 - 70) | 0.8 (0.6 - 1.0) | 50 (40 - 70) | 0.8 (0.6 - 1.0) | 50 (40 - 70) |
| Kuwait | 0.8 (0.6 - 1.0) | 25 (20 - 35) | 0.7 (0.6 - 1.0) | 25 (20 - 35) | 0.7 (0.6 - 1.0) | 25 (20 - 35) |
| Luxembourg | 0.8 (0.6 - 1.1) | 5 (4 - 7) | 0.8 (0.6 - 1.1) | 5 (4 - 7) | 0.8 (0.6 - 1.1) | 5 (4 - 7) |
| Moldova | 0.8 (0.6 - 1.1) | 30 (20 - 40) | 2.1 (1.5 - 3.0) | 80 (50 - 100) | 7.1 (4.9 - 10.3) | 250 (200 - 350) |
| Netherlands | 0.8 (0.6 - 1.1) | 150 (100 - 200) | 0.8 (0.6 - 1.2) | 150 (100 - 200) | 0.8 (0.6 - 1.2) | 150 (100 - 200) |
| Israel | 0.7 (0.5 - 1.0) | 60 (40 - 80) | 0.7 (0.5 - 1.0) | 60 (40 - 80) | 0.7 (0.5 - 1.0) | 60 (40 - 80) |
| Italy | 0.7 (0.6 - 0.9) | 450 (350 - 550) | 0.7 (0.6 - 1.0) | 450 (350 - 550) | 0.7 (0.6 - 1.0) | 450 (350 - 550) |
| Mongolia | 0.7 (0.7 - 0.8) | 25 (20 - 25) | 1.9 (1.8 - 2.0) | 60 (60 - 60) | 6.4 (5.9 - 6.9) | 200 (200 - 200) |
| Republic of Serbia | 0.7 (0.5 - 0.9) | 45 (35 - 60) | 1.7 (1.3 - 2.3) | 100 (90 - 150) | 5.9 (4.3 - 8.1) | 400 (300 - 550) |
| Switzerland | 0.7 (0.5 - 0.9) | 60 (45 - 80) | 0.7 (0.5 - 0.9) | 60 (45 - 80) | 0.7 (0.5 - 0.9) | 60 (45 - 80) |
| United Arab Emirates | 0.7 (0.5 - 0.9) | 60 (40 - 80) | 0.7 (0.5 - 1.0) | 60 (45 - 80) | 0.7 (0.5 - 1.0) | 60 (45 - 80) |
| Kazakhstan | 0.6 (0.6 - 0.6) | 100 (100 - 100) | 1.6 (1.5 - 1.7) | 300 (300 - 300) | 5.5 (5.1 - 5.8) | 1,000 (950 - 1,050) |
| Romania | 0.6 (0.5 - 0.8) | 200 (150 - 250) | 0.6 (0.5 - 0.8) | 200 (150 - 250) | 0.6 (0.5 - 0.8) | 200 (150 - 250) |
| Hungary | 0.5 (0.4 - 0.7) | 45 (35 - 60) | 0.5 (0.4 - 0.7) | 45 (35 - 60) | 0.5 (0.4 - 0.7) | 45 (35 - 60) |
| North Korea | 0.5 (0.3 - 0.6) | 100 (80 - 150) | 1.3 (0.9 - 1.7) | 300 (200 - 400) | 4.2 (3.2 - 5.8) | 950 (700 - 1,300) |
| San Marino | 0.5 (0.4 - 0.7) | 0 (0 - 0) | 0.6 (0.4 - 0.7) | 0 (0 - 0) | 0.6 (0.4 - 0.7) | 0 (0 - 0) |
| Singapore | 0.5 (0.3 - 0.8) | 20 (10 - 30) | 0.5 (0.3 - 0.8) | 20 (15 - 30) | 0.5 (0.3 - 0.8) | 20 (15 - 30) |
| Japan | 0.4 (0.3 - 0.6) | 500 (350 - 750) | 0.4 (0.3 - 0.6) | 500 (350 - 750) | 0.4 (0.3 - 0.6) | 500 (350 - 750) |
| Norway | 0.4 (0.3 - 0.6) | 20 (15 - 30) | 0.5 (0.3 - 0.6) | 20 (15 - 30) | 0.5 (0.3 - 0.6) | 20 (15 - 30) |
| Russia | 0.4 (0.3 - 0.4) | 500 (500 - 500) | 0.4 (0.3 - 0.4) | 500 (500 - 500) | 0.4 (0.3 - 0.4) | 500 (500 - 500) |
| South Korea | 0.4 (0.3 - 0.5) | 200 (150 - 250) | 0.4 (0.3 - 0.5) | 200 (150 - 250) | 0.4 (0.3 - 0.5) | 200 (150 - 250) |
| Sweden | 0.4 (0.3 - 0.5) | 40 (30 - 45) | 0.4 (0.3 - 0.5) | 40 (30 - 45) | 0.4 (0.3 - 0.5) | 40 (30 - 45) |
| Finland | 0.3 (0.3 - 0.4) | 20 (15 - 20) | 0.3 (0.3 - 0.4) | 20 (15 - 20) | 0.3 (0.3 - 0.4) | 20 (15 - 20) |
| CuraÃ§ao | 0.2 (0.2 - 0.3) | 0 (0 - 1) | 0.2 (0.1 - 0.3) | 0 (0 - 1) | 0.2 (0.1 - 0.3) | 0 (0 - 1) |
| Canada | 0.1 (0.1 - 0.1) | 20 (20 - 20) | 0.1 (0.1 - 0.1) | 20 (20 - 20) | 0.1 (0.1 - 0.1) | 20 (20 - 20) |

Please note that our country-level estimates (no correction for under‑reporting) are most appropriate for countries with low incidence rates and robust reporting systems. In contrast, the hospital-level estimates (partial correction for under‑reporting) and survey-level estimates (extensive correction for under‑reporting) are more suitable for countries with high incidence rates and/or inadequate surveillance infrastructure.

Table B: Estimated mortality incidence per 100,000 people and number of annual number of deaths (95% Predictive Interval) derived from country‑level estimates (reflecting no correction for under‑reporting), derived from hospital‑based estimates (reflecting partial correction for under‑reporting), and derived from community‑based survey estimates (reflecting extensive correction for under‑reporting). Please note that no corrections have been made for high-income countries (HICs) in hospital-based and survey-based estimates. *Estimates presented in the manuscript as low estimate and high estimate scenarios.

|  | **Country level estimates**  **(no correction for under‑reporting)** | | **Hospital level estimates**  **(partial correction for under‑reporting) *** | | **Survey level estimates**  **(extensive correction for under‑reporting) *** | |
| --- | --- | --- | --- | --- | --- | --- |
| **Country** | **Incidence/100,000** | **Annual envenomings** | **Incidence/100,000** | **Annual envenomings** | **Incidence/100,000** | **Annual envenomings** |
| Democratic Republic of the Congo | 1.1 (0.9 - 1.3) | 1,250 (1,050 - 1,500) | 18.2 (14.8 - 22.2) | 20,700 (16,800 - 25,300) | 34.0 (27.8 - 41.0) | 38,700 (31,600 - 46,600) |
| Mali | 1.1 (0.8 - 1.6) | 250 (200 - 400) | 18.7 (13.5 - 25.8) | 4,350 (3,150 - 6,000) | 34.8 (25.6 - 48.6) | 8,100 (6,000 - 11,300) |
| Burkina Faso | 1.0 (0.6 - 1.6) | 200 (150 - 350) | 16.2 (9.3 - 26.3) | 3,700 (2,150 - 6,000) | 30.5 (18.2 - 49.6) | 6,950 (4,150 - 11,300) |
| Yemen | 1.0 (0.7 - 1.4) | 300 (200 - 450) | 16.5 (11.5 - 24.1) | 4,900 (3,400 - 7,200) | 30.5 (21.5 - 45.3) | 9,100 (6,400 - 13,500) |
| Guinea-Bissau | 0.9 (0.4 - 1.7) | 15 (8 - 30) | 14.5 (7.2 - 26.8) | 250 (150 - 450) | 26.6 (13.5 - 49.7) | 450 (250 - 900) |
| Malawi | 0.9 (0.4 - 1.9) | 200 (80 - 350) | 15.3 (6.9 - 30.4) | 2,850 (1,300 - 5,650) | 29.4 (13.6 - 57.2) | 5,500 (2,550 - 10,700) |
| Afghanistan | 0.8 (0.6 - 1.1) | 250 (200 - 350) | 13.2 (10.0 - 18.3) | 4,000 (3,050 - 5,550) | 24.9 (18.7 - 34.7) | 7,550 (5,650 - 10,500) |
| Central African Republic | 0.8 (0.5 - 1.1) | 45 (30 - 60) | 12.9 (8.9 - 18.9) | 700 (500 - 1,050) | 24.2 (16.5 - 34.3) | 1,300 (900 - 1,850) |
| Guinea | 0.8 (0.4 - 1.3) | 100 (50 - 150) | 13.0 (7.0 - 22.6) | 1,600 (850 - 2,800) | 24.5 (12.7 - 41.8) | 3,000 (1,550 - 5,150) |
| Kenya | 0.8 (0.4 - 1.4) | 450 (200 - 750) | 13.1 (6.4 - 22.9) | 7,200 (3,500 - 12,600) | 24.6 (12.1 - 43.6) | 13,500 (6,650 - 24,000) |
| Nigeria | 0.8 (0.6 - 1.1) | 1,800 (1,350 - 2,350) | 13.8 (10.5 - 18.3) | 29,600 (22,700 - 39,300) | 25.7 (19.7 - 33.2) | 55,300 (42,400 - 71,500) |
| Republic of the Congo | 0.8 (0.5 - 1.4) | 30 (20 - 50) | 13.5 (7.6 - 21.5) | 500 (300 - 800) | 25.5 (14.8 - 41.8) | 950 (550 - 1,600) |
| Zambia | 0.8 (0.6 - 1.1) | 150 (100 - 200) | 13.4 (10.0 - 18.2) | 2,500 (1,850 - 3,400) | 25.0 (18.2 - 33.1) | 4,700 (3,400 - 6,200) |
| Belize | 0.7 (0.4 - 1.2) | 3 (2 - 5) | 12.3 (7.0 - 20.9) | 50 (30 - 90) | 22.9 (13.0 - 37.9) | 90 (50 - 150) |
| Pakistan | 0.7 (0.6 - 0.9) | 1,650 (1,300 - 2,100) | 11.6 (9.0 - 15.6) | 26,700 (20,600 - 36,000) | 22.0 (16.6 - 29.2) | 50,500 (38,200 - 67,100) |
| South Sudan | 0.7 (0.5 - 1.0) | 100 (90 - 150) | 11.9 (8.7 - 16.5) | 2,000 (1,450 - 2,750) | 22.1 (16.1 - 30.2) | 3,700 (2,700 - 5,050) |
| Uganda | 0.7 (0.3 - 1.2) | 300 (150 - 500) | 11.4 (5.5 - 20.5) | 4,850 (2,350 - 8,750) | 21.4 (10.4 - 39.0) | 9,100 (4,400 - 16,600) |
| Angola | 0.6 (0.5 - 0.8) | 200 (150 - 250) | 9.9 (7.8 - 12.8) | 3,500 (2,750 - 4,500) | 18.5 (14.6 - 24.0) | 6,550 (5,150 - 8,450) |
| Cameroon | 0.6 (0.3 - 0.9) | 150 (90 - 250) | 9.3 (4.8 - 15.8) | 2,600 (1,350 - 4,450) | 17.6 (9.1 - 29.6) | 4,950 (2,550 - 8,350) |
| Chad | 0.6 (0.5 - 0.7) | 90 (80 - 100) | 9.3 (7.5 - 11.6) | 1,550 (1,250 - 1,900) | 17.5 (14.3 - 21.9) | 2,900 (2,350 - 3,650) |
| Ethiopia | 0.6 (0.4 - 0.7) | 600 (450 - 750) | 9.1 (6.9 - 11.7) | 9,400 (7,200 - 12,100) | 17.0 (12.9 - 22.4) | 17,600 (13,400 - 23,200) |
| Gambia | 0.6 (0.3 - 1.2) | 15 (6 - 25) | 9.5 (4.8 - 18.5) | 200 (100 - 400) | 18.3 (8.7 - 39.7) | 400 (200 - 850) |
| Ivory Coast | 0.6 (0.2 - 1.4) | 150 (50 - 350) | 10.5 (3.6 - 24.0) | 2,700 (950 - 6,200) | 19.0 (6.5 - 42.2) | 4,950 (1,700 - 10,900) |
| Liberia | 0.6 (0.2 - 1.2) | 25 (10 - 50) | 9.6 (4.2 - 20.6) | 400 (150 - 850) | 17.7 (7.4 - 38.3) | 750 (300 - 1,600) |
| Myanmar | 0.6 (0.4 - 0.9) | 300 (150 - 450) | 9.7 (6.4 - 14.7) | 4,600 (3,050 - 6,950) | 18.2 (11.9 - 26.9) | 8,650 (5,650 - 12,800) |
| Niger | 0.6 (0.5 - 0.8) | 150 (100 - 200) | 9.8 (7.8 - 12.3) | 2,350 (1,850 - 2,950) | 18.2 (14.6 - 22.9) | 4,350 (3,450 - 5,450) |
| United Republic of Tanzania | 0.6 (0.5 - 0.9) | 350 (250 - 500) | 10.5 (7.3 - 14.5) | 5,850 (4,100 - 8,100) | 19.7 (13.5 - 27.8) | 11,000 (7,550 - 15,500) |
| Zimbabwe | 0.6 (0.4 - 0.9) | 90 (60 - 150) | 10.2 (6.9 - 15.2) | 1,450 (1,000 - 2,150) | 19.1 (12.6 - 27.9) | 2,750 (1,800 - 4,000) |
| Mauritania | 0.5 (0.4 - 0.7) | 25 (20 - 30) | 8.9 (7.0 - 11.3) | 400 (300 - 500) | 16.5 (13.0 - 21.0) | 700 (550 - 900) |
| Mexico | 0.5 (0.4 - 0.7) | 750 (600 - 950) | 8.6 (7.0 - 10.9) | 12,000 (9,800 - 15,200) | 16.1 (12.8 - 20.2) | 22,500 (17,900 - 28,200) |
| Mozambique | 0.5 (0.3 - 0.8) | 150 (100 - 250) | 9.0 (5.3 - 13.8) | 2,750 (1,600 - 4,200) | 16.9 (10.4 - 25.6) | 5,100 (3,150 - 7,750) |
| Papua New Guinea | 0.5 (0.3 - 0.7) | 40 (25 - 60) | 7.3 (4.7 - 11.4) | 650 (400 - 1,000) | 13.7 (9.0 - 22.0) | 1,200 (800 - 1,950) |
| Senegal | 0.5 (0.3 - 0.8) | 80 (50 - 150) | 8.0 (5.1 - 12.9) | 1,300 (800 - 2,100) | 15.0 (9.3 - 24.0) | 2,400 (1,500 - 3,850) |
| Sierra Leone | 0.5 (0.2 - 0.9) | 30 (15 - 50) | 7.7 (3.4 - 14.3) | 450 (200 - 850) | 14.4 (6.6 - 28.0) | 900 (400 - 1,700) |
| Venezuela | 0.5 (0.4 - 0.6) | 150 (100 - 200) | 7.8 (6.1 - 10.3) | 2,450 (1,850 - 3,200) | 14.8 (11.4 - 19.4) | 4,550 (3,500 - 6,000) |
| Benin | 0.4 (0.2 - 0.7) | 50 (30 - 90) | 6.5 (3.6 - 11.4) | 850 (450 - 1,500) | 12.2 (6.4 - 20.2) | 1,600 (850 - 2,650) |
| Burundi | 0.4 (0.2 - 0.7) | 45 (20 - 80) | 6.2 (2.9 - 11.3) | 700 (350 - 1,300) | 11.9 (5.6 - 22.5) | 1,350 (650 - 2,550) |
| Cambodia | 0.4 (0.2 - 0.9) | 80 (30 - 150) | 7.1 (2.8 - 13.7) | 1,400 (550 - 2,650) | 13.5 (5.3 - 27.6) | 2,600 (1,050 - 5,350) |
| Djibouti | 0.4 (0.2 - 0.7) | 4 (2 - 7) | 6.3 (3.4 - 11.6) | 60 (35 - 100) | 11.6 (6.3 - 21.5) | 100 (60 - 200) |
| Eritrea | 0.4 (0.2 - 1.0) | 20 (8 - 45) | 7.3 (3.2 - 15.7) | 300 (150 - 650) | 13.4 (5.9 - 29.2) | 550 (250 - 1,250) |
| Guatemala | 0.4 (0.2 - 0.8) | 80 (45 - 150) | 7.1 (4.0 - 12.9) | 1,300 (750 - 2,350) | 13.5 (7.9 - 23.4) | 2,500 (1,450 - 4,300) |
| Guyana | 0.4 (0.2 - 0.6) | 3 (2 - 4) | 0.4 (0.2 - 0.6) | 3 (2 - 4) | 0.4 (0.2 - 0.6) | 3 (2 - 4) |
| Somalia | 0.4 (0.2 - 0.6) | 30 (20 - 50) | 6.1 (3.6 - 10.2) | 500 (300 - 850) | 11.4 (6.5 - 19.1) | 900 (500 - 1,550) |
| Somaliland | 0.4 (0.2 - 0.7) | 10 (7 - 20) | 6.7 (4.1 - 11.2) | 200 (100 - 300) | 12.3 (7.6 - 20.2) | 350 (200 - 550) |
| Sudan | 0.4 (0.4 - 0.5) | 200 (150 - 200) | 6.9 (5.9 - 8.2) | 2,950 (2,500 - 3,450) | 12.9 (11.0 - 15.3) | 5,500 (4,700 - 6,550) |
| Botswana | 0.3 (0.2 - 0.4) | 7 (5 - 9) | 4.6 (3.4 - 6.4) | 100 (80 - 150) | 8.6 (6.3 - 11.8) | 200 (150 - 300) |
| Equatorial Guinea | 0.3 (0.1 - 0.8) | 3 (1 - 9) | 4.6 (1.2 - 11.9) | 50 (15 - 150) | 9.0 (2.3 - 26.2) | 100 (25 - 300) |
| eSwatini | 0.3 (0.2 - 0.5) | 3 (2 - 6) | 5.1 (2.4 - 8.6) | 50 (25 - 90) | 9.5 (4.6 - 16.2) | 100 (50 - 150) |
| Gabon | 0.3 (0.1 - 0.7) | 8 (2 - 20) | 5.0 (1.4 - 11.9) | 150 (40 - 350) | 9.3 (2.6 - 23.1) | 250 (70 - 650) |
| Indonesia | 0.3 (0.2 - 0.4) | 700 (550 - 1,000) | 4.4 (3.1 - 6.1) | 11,900 (8,550 - 16,400) | 8.1 (6.1 - 11.5) | 22,100 (16,600 - 31,100) |
| Laos | 0.3 (0.1 - 0.5) | 20 (9 - 40) | 4.3 (2.0 - 8.4) | 300 (150 - 650) | 8.0 (3.7 - 14.6) | 600 (300 - 1,100) |
| Namibia | 0.3 (0.2 - 0.4) | 8 (6 - 10) | 5.0 (3.8 - 6.6) | 100 (90 - 150) | 9.3 (6.9 - 12.5) | 250 (150 - 300) |
| Nepal | 0.3 (0.2 - 0.5) | 100 (70 - 200) | 4.7 (2.8 - 8.1) | 1,950 (1,150 - 3,350) | 8.7 (5.1 - 15.2) | 3,650 (2,150 - 6,300) |
| SÃ£o TomÃ© and Principe | 0.3 (0.0 - 1.0) | 0 (0 - 2) | 4.2 (0.7 - 15.3) | 6 (1 - 25) | 7.9 (1.2 - 30.7) | 10 (2 - 45) |
| Bangladesh | 0.2 (0.1 - 0.3) | 250 (100 - 450) | 2.5 (1.2 - 4.7) | 4,000 (1,900 - 7,500) | 4.7 (2.2 - 8.6) | 7,550 (3,550 - 13,800) |
| Brazil | 0.2 (0.2 - 0.3) | 500 (450 - 550) | 3.8 (3.5 - 4.2) | 8,000 (7,250 - 8,900) | 7.2 (6.4 - 8.0) | 15,000 (13,500 - 16,800) |
| Egypt | 0.2 (0.1 - 0.2) | 150 (150 - 200) | 2.7 (2.2 - 3.4) | 2,550 (2,050 - 3,250) | 5.1 (4.1 - 6.4) | 4,800 (3,900 - 6,000) |
| Ghana | 0.2 (0.1 - 0.4) | 70 (35 - 100) | 3.4 (1.7 - 5.9) | 1,100 (550 - 1,900) | 6.5 (3.2 - 11.2) | 2,100 (1,050 - 3,650) |
| India | 0.2 (0.1 - 0.2) | 2,300 (2,000 - 2,650) | 2.7 (2.3 - 3.2) | 37,600 (32,000 - 44,200) | 5.0 (4.2 - 5.9) | 70,100 (58,800 - 82,500) |
| Morocco | 0.2 (0.1 - 0.3) | 60 (40 - 90) | 2.8 (1.8 - 4.3) | 1,000 (650 - 1,550) | 5.4 (3.5 - 8.7) | 1,950 (1,250 - 3,100) |
| Nicaragua | 0.2 (0.1 - 0.3) | 10 (6 - 15) | 2.6 (1.5 - 4.3) | 200 (100 - 300) | 4.9 (2.8 - 8.3) | 350 (200 - 550) |
| Panama | 0.2 (0.1 - 0.4) | 10 (6 - 15) | 0.2 (0.1 - 0.4) | 9 (6 - 15) | 0.2 (0.1 - 0.4) | 10 (5 - 15) |
| Paraguay | 0.2 (0.1 - 0.2) | 10 (8 - 20) | 2.7 (1.9 - 3.9) | 200 (150 - 300) | 5.1 (3.5 - 7.2) | 350 (250 - 550) |
| Rwanda | 0.2 (0.1 - 0.3) | 20 (9 - 40) | 2.6 (1.2 - 5.1) | 350 (150 - 700) | 4.9 (2.0 - 9.3) | 650 (250 - 1,250) |
| Suriname | 0.2 (0.1 - 0.4) | 1 (1 - 3) | 3.5 (1.4 - 7.1) | 20 (8 - 45) | 6.5 (2.5 - 13.2) | 40 (15 - 80) |
| Togo | 0.2 (0.1 - 0.5) | 20 (10 - 40) | 4.0 (1.9 - 7.2) | 350 (150 - 600) | 7.6 (3.5 - 13.6) | 650 (300 - 1,150) |
| Algeria | 0.1 (0.1 - 0.1) | 40 (35 - 50) | 1.6 (1.4 - 1.9) | 700 (600 - 800) | 3.0 (2.5 - 3.5) | 1,300 (1,100 - 1,550) |
| Argentina | 0.1 (0.1 - 0.1) | 30 (25 - 35) | 1.1 (0.9 - 1.4) | 500 (400 - 600) | 2.1 (1.7 - 2.5) | 950 (800 - 1,150) |
| Bhutan | 0.1 (0.0 - 0.1) | 1 (0 - 1) | 0.9 (0.3 - 1.8) | 8 (3 - 15) | 1.6 (0.6 - 3.4) | 15 (6 - 30) |
| Bolivia | 0.1 (0.1 - 0.2) | 15 (15 - 25) | 2.4 (1.8 - 3.2) | 300 (200 - 400) | 4.5 (3.3 - 6.0) | 550 (400 - 700) |
| Colombia | 0.1 (0.1 - 0.2) | 90 (70 - 100) | 2.3 (1.8 - 3.1) | 1,450 (1,100 - 1,900) | 4.4 (3.3 - 5.7) | 2,750 (2,050 - 3,550) |
| Costa Rica | 0.1 (0.1 - 0.3) | 7 (3 - 15) | 0.1 (0.1 - 0.3) | 7 (4 - 10) | 0.2 (0.1 - 0.3) | 7 (4 - 15) |
| East Timor | 0.1 (0.0 - 0.3) | 1 (1 - 4) | 1.7 (0.7 - 4.0) | 25 (9 - 60) | 3.2 (1.3 - 7.1) | 45 (20 - 100) |
| Ecuador | 0.1 (0.0 - 0.1) | 15 (7 - 25) | 1.2 (0.6 - 2.3) | 200 (100 - 400) | 2.3 (1.2 - 4.3) | 400 (200 - 750) |
| Honduras | 0.1 (0.1 - 0.3) | 15 (7 - 25) | 2.3 (1.3 - 3.9) | 200 (100 - 350) | 4.3 (2.2 - 7.7) | 400 (200 - 750) |
| Hong Kong S.A.R. | 0.1 (0.0 - 0.1) | 4 (2 - 9) | 0.1 (0.0 - 0.1) | 4 (2 - 9) | 0.1 (0.0 - 0.1) | 4 (2 - 8) |
| Iran | 0.1 (0.1 - 0.1) | 60 (45 - 80) | 1.2 (1.0 - 1.6) | 1,000 (750 - 1,250) | 2.3 (1.8 - 2.9) | 1,850 (1,400 - 2,350) |
| Iraq | 0.1 (0.1 - 0.2) | 50 (35 - 80) | 1.9 (1.3 - 2.7) | 850 (600 - 1,200) | 3.5 (2.4 - 5.2) | 1,550 (1,100 - 2,350) |
| Jordan | 0.1 (0.0 - 0.2) | 6 (3 - 15) | 1.5 (0.6 - 2.9) | 100 (45 - 200) | 2.7 (1.2 - 5.6) | 200 (80 - 400) |
| Kyrgyzstan | 0.1 (0.0 - 0.1) | 4 (2 - 7) | 1.1 (0.7 - 2.0) | 70 (40 - 100) | 2.1 (1.3 - 3.7) | 100 (80 - 200) |
| Lesotho | 0.1 (0.0 - 0.1) | 1 (0 - 2) | 0.9 (0.3 - 1.8) | 15 (6 - 35) | 1.6 (0.6 - 3.3) | 30 (10 - 60) |
| Palestine | 0.1 (0.0 - 0.1) | 4 (1 - 9) | 1.1 (0.3 - 2.5) | 70 (20 - 150) | 2.0 (0.7 - 4.5) | 150 (45 - 300) |
| Peru | 0.1 (0.1 - 0.2) | 45 (35 - 60) | 2.2 (1.7 - 2.8) | 750 (600 - 1,000) | 4.1 (3.2 - 5.2) | 1,450 (1,100 - 1,850) |
| Philippines | 0.1 (0.0 - 0.2) | 80 (40 - 150) | 1.4 (0.6 - 2.7) | 1,350 (650 - 2,600) | 2.5 (1.2 - 5.3) | 2,500 (1,250 - 5,150) |
| Sri Lanka | 0.1 (0.1 - 0.2) | 25 (15 - 45) | 2.3 (1.2 - 3.8) | 450 (250 - 750) | 4.2 (2.2 - 7.0) | 850 (450 - 1,400) |
| Syria | 0.1 (0.0 - 0.1) | 20 (10 - 35) | 1.3 (0.7 - 2.3) | 350 (150 - 600) | 2.3 (1.2 - 4.2) | 600 (300 - 1,100) |
| Tajikistan | 0.1 (0.0 - 0.2) | 8 (4 - 15) | 1.4 (0.8 - 2.7) | 150 (70 - 250) | 2.5 (1.4 - 4.7) | 250 (150 - 450) |
| Thailand | 0.1 (0.1 - 0.2) | 90 (60 - 150) | 2.0 (1.3 - 3.0) | 1,500 (950 - 2,200) | 3.8 (2.4 - 5.9) | 2,800 (1,750 - 4,400) |
| Uruguay | 0.1 (0.0 - 0.1) | 2 (1 - 3) | 0.1 (0.0 - 0.1) | 2 (1 - 3) | 0.1 (0.0 - 0.1) | 2 (1 - 3) |
| Uzbekistan | 0.1 (0.0 - 0.1) | 25 (15 - 40) | 1.2 (0.8 - 1.8) | 400 (250 - 600) | 2.2 (1.5 - 3.6) | 750 (500 - 1,200) |
| Vietnam | 0.1 (0.1 - 0.3) | 150 (70 - 250) | 2.4 (1.1 - 4.5) | 2,350 (1,100 - 4,350) | 4.6 (2.1 - 8.6) | 4,400 (2,000 - 8,250) |
| Western Sahara | 0.1 (0.0 - 0.5) | 0 (0 - 0) | 2.3 (0.6 - 7.0) | 1 (0 - 4) | 4.7 (1.2 - 14.5) | 3 (1 - 9) |
| Albania | 0.0 (0.0 - 0.1) | 1 (0 - 2) | 0.5 (0.2 - 0.9) | 15 (5 - 25) | 0.9 (0.3 - 1.7) | 25 (9 - 45) |
| Andorra | 0.0 (0.0 - 0.0) | 0 (0 - 0) | 0.0 (0.0 - 0.0) | 0 (0 - 0) | 0.0 (0.0 - 0.0) | 0 (0 - 0) |
| Armenia | 0.0 (0.0 - 0.1) | 1 (0 - 2) | 0.5 (0.2 - 1.1) | 15 (6 - 30) | 0.9 (0.4 - 1.9) | 25 (10 - 50) |
| Australia | 0.0 (0.0 - 0.0) | 1 (1 - 1) | 0.0 (0.0 - 0.0) | 1 (1 - 1) | 0.0 (0.0 - 0.0) | 1 (1 - 1) |
| Austria | 0.0 (0.0 - 0.0) | 0 (0 - 1) | 0.0 (0.0 - 0.0) | 0 (0 - 1) | 0.0 (0.0 - 0.0) | 0 (0 - 1) |
| Azerbaijan | 0.0 (0.0 - 0.1) | 3 (2 - 7) | 0.5 (0.3 - 1.1) | 50 (25 - 100) | 1.0 (0.5 - 2.2) | 100 (50 - 250) |
| Bahrain | 0.0 (0.0 - 0.0) | 0 (0 - 0) | 0.0 (0.0 - 0.0) | 0 (0 - 0) | 0.0 (0.0 - 0.0) | 0 (0 - 0) |
| Belarus | 0.0 (0.0 - 0.0) | 1 (0 - 1) | 0.1 (0.1 - 0.2) | 15 (7 - 20) | 0.3 (0.1 - 0.5) | 25 (15 - 40) |
| Belgium | 0.0 (0.0 - 0.0) | 0 (0 - 1) | 0.0 (0.0 - 0.0) | 0 (0 - 1) | 0.0 (0.0 - 0.0) | 0 (0 - 1) |
| Bosnia and Herzegovina | 0.0 (0.0 - 0.0) | 0 (0 - 1) | 0.2 (0.0 - 0.5) | 7 (1 - 20) | 0.4 (0.1 - 0.9) | 10 (2 - 30) |
| Brunei | 0.0 (0.0 - 0.0) | 0 (0 - 0) | 0.0 (0.0 - 0.0) | 0 (0 - 0) | 0.0 (0.0 - 0.0) | 0 (0 - 0) |
| Bulgaria | 0.0 (0.0 - 0.0) | 1 (0 - 1) | 0.0 (0.0 - 0.0) | 1 (0 - 2) | 0.0 (0.0 - 0.0) | 1 (0 - 2) |
| Canada | 0.0 (0.0 - 0.0) | 0 (0 - 0) | 0.0 (0.0 - 0.0) | 0 (0 - 0) | 0.0 (0.0 - 0.0) | 0 (0 - 0) |
| China | 0.0 (0.0 - 0.0) | 600 (550 - 700) | 0.7 (0.6 - 0.8) | 10,200 (9,200 - 11,300) | 1.3 (1.2 - 1.5) | 19,000 (17,200 - 21,000) |
| Croatia | 0.0 (0.0 - 0.0) | 0 (0 - 1) | 0.0 (0.0 - 0.0) | 0 (0 - 1) | 0.0 (0.0 - 0.0) | 0 (0 - 1) |
| CuraÃ§ao | 0.0 (0.0 - 0.0) | 0 (0 - 0) | 0.0 (0.0 - 0.0) | 0 (0 - 0) | 0.0 (0.0 - 0.0) | 0 (0 - 0) |
| Cyprus | 0.0 (0.0 - 0.0) | 0 (0 - 0) | 0.0 (0.0 - 0.0) | 0 (0 - 0) | 0.0 (0.0 - 0.0) | 0 (0 - 0) |
| Czechia | 0.0 (0.0 - 0.0) | 0 (0 - 1) | 0.0 (0.0 - 0.0) | 0 (0 - 1) | 0.0 (0.0 - 0.0) | 0 (0 - 1) |
| Denmark | 0.0 (0.0 - 0.0) | 0 (0 - 1) | 0.0 (0.0 - 0.0) | 0 (0 - 1) | 0.0 (0.0 - 0.0) | 0 (0 - 1) |
| El Salvador | 0.0 (0.0 - 0.1) | 2 (1 - 5) | 0.6 (0.2 - 1.2) | 40 (10 - 80) | 1.0 (0.3 - 2.2) | 70 (25 - 150) |
| Estonia | 0.0 (0.0 - 0.0) | 0 (0 - 0) | 0.0 (0.0 - 0.0) | 0 (0 - 0) | 0.0 (0.0 - 0.0) | 0 (0 - 0) |
| Finland | 0.0 (0.0 - 0.0) | 1 (1 - 2) | 0.0 (0.0 - 0.0) | 1 (1 - 1) | 0.0 (0.0 - 0.0) | 1 (1 - 1) |
| France | 0.0 (0.0 - 0.0) | 5 (2 - 8) | 0.0 (0.0 - 0.0) | 5 (2 - 8) | 0.0 (0.0 - 0.0) | 5 (2 - 8) |
| French Guiana | 0.0 (0.0 - 0.1) | 0 (0 - 0) | 0.0 (0.0 - 0.1) | 0 (0 - 0) | 0.0 (0.0 - 0.1) | 0 (0 - 0) |
| Georgia | 0.0 (0.0 - 0.1) | 1 (1 - 3) | 0.5 (0.3 - 1.1) | 20 (10 - 40) | 1.0 (0.5 - 2.1) | 40 (20 - 80) |
| Germany | 0.0 (0.0 - 0.0) | 3 (2 - 5) | 0.0 (0.0 - 0.0) | 4 (2 - 5) | 0.0 (0.0 - 0.0) | 3 (2 - 5) |
| Greece | 0.0 (0.0 - 0.0) | 1 (0 - 2) | 0.0 (0.0 - 0.0) | 1 (0 - 3) | 0.0 (0.0 - 0.0) | 1 (0 - 3) |
| Hungary | 0.0 (0.0 - 0.0) | 0 (0 - 1) | 0.0 (0.0 - 0.0) | 0 (0 - 1) | 0.0 (0.0 - 0.0) | 0 (0 - 1) |
| Israel | 0.0 (0.0 - 0.0) | 1 (0 - 1) | 0.0 (0.0 - 0.0) | 1 (0 - 1) | 0.0 (0.0 - 0.0) | 1 (0 - 1) |
| Italy | 0.0 (0.0 - 0.0) | 4 (2 - 8) | 0.0 (0.0 - 0.0) | 4 (2 - 8) | 0.0 (0.0 - 0.0) | 4 (2 - 8) |
| Japan | 0.0 (0.0 - 0.0) | 8 (3 - 20) | 0.0 (0.0 - 0.0) | 8 (3 - 20) | 0.0 (0.0 - 0.0) | 8 (4 - 20) |
| Kazakhstan | 0.0 (0.0 - 0.0) | 1 (1 - 1) | 0.1 (0.1 - 0.1) | 15 (15 - 20) | 0.2 (0.1 - 0.2) | 30 (25 - 35) |
| Kosovo | 0.0 (0.0 - 0.0) | 0 (0 - 1) | 0.3 (0.1 - 0.7) | 6 (2 - 15) | 0.5 (0.2 - 1.1) | 10 (4 - 25) |
| Kuwait | 0.0 (0.0 - 0.0) | 0 (0 - 0) | 0.0 (0.0 - 0.0) | 0 (0 - 0) | 0.0 (0.0 - 0.0) | 0 (0 - 0) |
| Latvia | 0.0 (0.0 - 0.0) | 0 (0 - 1) | 0.0 (0.0 - 0.0) | 0 (0 - 1) | 0.0 (0.0 - 0.0) | 0 (0 - 1) |
| Lebanon | 0.0 (0.0 - 0.1) | 3 (1 - 8) | 0.6 (0.2 - 1.5) | 50 (15 - 150) | 1.1 (0.3 - 2.7) | 100 (30 - 250) |
| Libya | 0.0 (0.0 - 0.0) | 2 (2 - 3) | 0.5 (0.4 - 0.6) | 35 (30 - 40) | 0.9 (0.8 - 1.1) | 60 (50 - 80) |
| Liechtenstein | 0.0 (0.0 - 0.0) | 0 (0 - 0) | 0.0 (0.0 - 0.0) | 0 (0 - 0) | 0.0 (0.0 - 0.0) | 0 (0 - 0) |
| Lithuania | 0.0 (0.0 - 0.0) | 0 (0 - 1) | 0.0 (0.0 - 0.0) | 0 (0 - 1) | 0.0 (0.0 - 0.0) | 0 (0 - 1) |
| Luxembourg | 0.0 (0.0 - 0.0) | 0 (0 - 0) | 0.0 (0.0 - 0.0) | 0 (0 - 0) | 0.0 (0.0 - 0.0) | 0 (0 - 0) |
| Macao S.A.R | 0.0 (0.0 - 0.1) | 0 (0 - 0) | 0.0 (0.0 - 0.0) | 0 (0 - 0) | 0.0 (0.0 - 0.0) | 0 (0 - 0) |
| Malaysia | 0.0 (0.0 - 0.0) | 7 (4 - 15) | 0.4 (0.2 - 0.7) | 100 (60 - 200) | 0.7 (0.4 - 1.3) | 250 (100 - 450) |
| Martinique (France) | 0.0 (0.0 - 0.0) | 0 (0 - 0) | 0.0 (0.0 - 0.0) | 0 (0 - 0) | 0.0 (0.0 - 0.0) | 0 (0 - 0) |
| Moldova | 0.0 (0.0 - 0.0) | 0 (0 - 0) | 0.1 (0.0 - 0.2) | 4 (2 - 8) | 0.2 (0.1 - 0.4) | 7 (3 - 15) |
| Mongolia | 0.0 (0.0 - 0.0) | 0 (0 - 0) | 0.2 (0.1 - 0.2) | 5 (4 - 6) | 0.3 (0.2 - 0.4) | 9 (8 - 10) |
| Montenegro | 0.0 (0.0 - 0.1) | 0 (0 - 0) | 0.4 (0.1 - 0.9) | 2 (1 - 5) | 0.7 (0.2 - 1.7) | 4 (1 - 10) |
| Netherlands | 0.0 (0.0 - 0.0) | 1 (0 - 1) | 0.0 (0.0 - 0.0) | 1 (0 - 2) | 0.0 (0.0 - 0.0) | 1 (0 - 2) |
| North Korea | 0.0 (0.0 - 0.1) | 8 (4 - 15) | 0.5 (0.3 - 1.0) | 100 (70 - 250) | 1.0 (0.5 - 1.9) | 250 (100 - 450) |
| North Macedonia | 0.0 (0.0 - 0.0) | 0 (0 - 1) | 0.3 (0.1 - 0.6) | 7 (3 - 15) | 0.6 (0.3 - 1.2) | 10 (6 - 25) |
| Northern Cyprus | 0.0 (0.0 - 0.0) | 0 (0 - 0) | 0.0 (0.0 - 0.0) | 0 (0 - 0) | 0.0 (0.0 - 0.0) | 0 (0 - 0) |
| Norway | 0.0 (0.0 - 0.0) | 0 (0 - 1) | 0.0 (0.0 - 0.0) | 0 (0 - 1) | 0.0 (0.0 - 0.0) | 0 (0 - 1) |
| Oman | 0.0 (0.0 - 0.0) | 0 (0 - 0) | 0.0 (0.0 - 0.0) | 0 (0 - 0) | 0.0 (0.0 - 0.0) | 0 (0 - 0) |
| Poland | 0.0 (0.0 - 0.0) | 1 (1 - 3) | 0.0 (0.0 - 0.0) | 1 (1 - 2) | 0.0 (0.0 - 0.0) | 1 (1 - 2) |
| Portugal | 0.0 (0.0 - 0.0) | 1 (0 - 4) | 0.0 (0.0 - 0.0) | 1 (0 - 4) | 0.0 (0.0 - 0.0) | 1 (0 - 4) |
| Qatar | 0.0 (0.0 - 0.0) | 0 (0 - 0) | 0.0 (0.0 - 0.0) | 0 (0 - 0) | 0.0 (0.0 - 0.0) | 0 (0 - 0) |
| Republic of Serbia | 0.0 (0.0 - 0.0) | 0 (0 - 1) | 0.1 (0.0 - 0.2) | 6 (2 - 15) | 0.2 (0.1 - 0.4) | 10 (4 - 25) |
| Romania | 0.0 (0.0 - 0.0) | 1 (0 - 2) | 0.0 (0.0 - 0.0) | 1 (0 - 2) | 0.0 (0.0 - 0.0) | 1 (0 - 3) |
| Russia | 0.0 (0.0 - 0.0) | 10 (9 - 10) | 0.0 (0.0 - 0.0) | 10 (9 - 10) | 0.0 (0.0 - 0.0) | 10 (9 - 10) |
| Saint Lucia | 0.0 (0.0 - 0.2) | 0 (0 - 0) | 0.7 (0.1 - 2.7) | 1 (0 - 4) | 1.3 (0.1 - 5.2) | 2 (0 - 7) |
| San Marino | 0.0 (0.0 - 0.0) | 0 (0 - 0) | 0.0 (0.0 - 0.0) | 0 (0 - 0) | 0.0 (0.0 - 0.0) | 0 (0 - 0) |
| Saudi Arabia | 0.0 (0.0 - 0.0) | 3 (3 - 4) | 0.0 (0.0 - 0.0) | 3 (3 - 4) | 0.0 (0.0 - 0.0) | 3 (3 - 4) |
| Singapore | 0.0 (0.0 - 0.0) | 0 (0 - 0) | 0.0 (0.0 - 0.0) | 0 (0 - 0) | 0.0 (0.0 - 0.0) | 0 (0 - 0) |
| Slovakia | 0.0 (0.0 - 0.0) | 0 (0 - 1) | 0.0 (0.0 - 0.0) | 0 (0 - 1) | 0.0 (0.0 - 0.0) | 0 (0 - 1) |
| Slovenia | 0.0 (0.0 - 0.0) | 0 (0 - 0) | 0.0 (0.0 - 0.0) | 0 (0 - 0) | 0.0 (0.0 - 0.0) | 0 (0 - 0) |
| South Africa | 0.0 (0.0 - 0.1) | 30 (20 - 40) | 0.8 (0.5 - 1.1) | 450 (300 - 700) | 1.5 (1.0 - 2.2) | 900 (600 - 1,350) |
| South Korea | 0.0 (0.0 - 0.0) | 3 (2 - 6) | 0.0 (0.0 - 0.0) | 3 (2 - 6) | 0.0 (0.0 - 0.0) | 3 (2 - 6) |
| Spain | 0.0 (0.0 - 0.0) | 6 (3 - 10) | 0.0 (0.0 - 0.0) | 6 (3 - 10) | 0.0 (0.0 - 0.0) | 6 (4 - 10) |
| Sweden | 0.0 (0.0 - 0.0) | 1 (1 - 2) | 0.0 (0.0 - 0.0) | 1 (1 - 2) | 0.0 (0.0 - 0.0) | 1 (1 - 2) |
| Switzerland | 0.0 (0.0 - 0.0) | 0 (0 - 1) | 0.0 (0.0 - 0.0) | 0 (0 - 1) | 0.0 (0.0 - 0.0) | 0 (0 - 1) |
| Taiwan | 0.0 (0.0 - 0.1) | 10 (4 - 25) | 0.0 (0.0 - 0.1) | 10 (4 - 25) | 0.0 (0.0 - 0.1) | 10 (4 - 25) |
| Trinidad and Tobago | 0.0 (0.0 - 0.0) | 0 (0 - 0) | 0.0 (0.0 - 0.0) | 0 (0 - 0) | 0.0 (0.0 - 0.0) | 0 (0 - 0) |
| Tunisia | 0.0 (0.0 - 0.1) | 5 (3 - 8) | 0.8 (0.5 - 1.4) | 90 (50 - 150) | 1.4 (0.9 - 2.4) | 150 (100 - 250) |
| Turkey | 0.0 (0.0 - 0.0) | 15 (8 - 20) | 0.3 (0.2 - 0.4) | 200 (150 - 300) | 0.5 (0.3 - 0.7) | 400 (250 - 550) |
| Turkmenistan | 0.0 (0.0 - 0.1) | 3 (3 - 5) | 0.6 (0.4 - 0.8) | 60 (40 - 80) | 1.1 (0.8 - 1.7) | 100 (80 - 150) |
| Ukraine | 0.0 (0.0 - 0.0) | 6 (4 - 8) | 0.2 (0.2 - 0.3) | 100 (70 - 150) | 0.4 (0.3 - 0.6) | 200 (150 - 250) |
| United Arab Emirates | 0.0 (0.0 - 0.0) | 0 (0 - 0) | 0.0 (0.0 - 0.0) | 0 (0 - 0) | 0.0 (0.0 - 0.0) | 0 (0 - 0) |
| United Kingdom | 0.0 (0.0 - 0.0) | 5 (2 - 10) | 0.0 (0.0 - 0.0) | 5 (2 - 10) | 0.0 (0.0 - 0.0) | 5 (2 - 10) |
| United States of America | 0.0 (0.0 - 0.0) | 35 (30 - 35) | 0.0 (0.0 - 0.0) | 35 (30 - 35) | 0.0 (0.0 - 0.0) | 35 (30 - 35) |

Please note that our country-level estimates (no correction for under‑reporting) are most appropriate for countries with low incidence rates and robust reporting systems. In contrast, the hospital-level estimates (partial correction for under‑reporting) and survey-level estimates (extensive correction for under‑reporting) are more suitable for countries with high incidence rates and/or inadequate surveillance infrastructure.
